# Cyclin D1-CDK4 activity drives sensitivity to bortezomib in mantle cell lymphoma by blocking autophagy-mediated proteolysis of NOXA

**DOI:** 10.1186/s13045-018-0657-6

**Published:** 2018-09-04

**Authors:** Simon Heine, Markus Kleih, Neus Giménez, Kathrin Böpple, German Ott, Dolors Colomer, Walter E. Aulitzky, Heiko van der Kuip, Elisabeth Silkenstedt

**Affiliations:** 1 Dr. Margarete Fischer-Bosch-Institute of Clinical Pharmacology, Stuttgart, Germany; 2 Hematopathology Unit, Hospital Clínic – Institut d’Investigacions Biomèdiques August Pi i Sunyer (IDIBAPS), CIBERONC, Barcelona, Spain; 3 Department of Clinical Pathology, Robert-Bosch-Hospital, Stuttgart, Germany; 4 Department of Hematology and Oncology, Robert-Bosch-Hospital, Stuttgart, Germany; 5 LMU Klinikum der Universität München, Med. Klinik und Poliklinik III, Munich, Germany; 6 University of Tübingen, Tübingen, Germany

**Keywords:** Mantle cell lymphoma, Bortezomib, NOXA, CDK4, Autophagy

## Abstract

**Background:**

Mantle cell lymphoma (MCL) is an aggressive B-non-Hodgkin lymphoma with generally poor outcome. MCL is characterized by an aberrantly high cyclin D1-driven CDK4 activity. New molecular targeted therapies such as inhibitors of the ubiquitin-proteasome system (UPS) have shown promising results in preclinical studies and MCL patients. Our previous research revealed stabilization of the short-lived pro-apoptotic NOXA as a critical determinant for sensitivity to these inhibitors. It is currently unclear how cyclin D1 overexpression and aberrant CDK4 activity affect NOXA stabilization and treatment efficacy of UPS inhibitors in MCL.

**Methods:**

The effect of cyclin D1-driven CDK4 activity on response of MCL cell lines and primary cells to proteasome inhibitor treatment was investigated using survival assays (Flow cytometry, AnnexinV/PI) and Western blot analysis of NOXA protein. Half-life of NOXA protein was determined by cycloheximide treatment and subsequent Western blot analysis. The role of autophagy was analyzed by LC3-II protein expression and autophagolysosome detection. Furthermore, silencing of autophagy-related genes was performed using siRNA and MCL cells were treated with autophagy inhibitors in combination with proteasome and CDK4 inhibition.

**Results:**

In this study, we show that proteasome inhibitor-mediated cell death in MCL depends on cyclin D1-driven CDK4 activity. Inhibition of cyclin D1/CDK4 activity significantly reduced proteasome inhibitor-mediated stabilization of NOXA protein, mainly driven by an autophagy-mediated proteolysis. Bortezomib-induced cell death was significantly potentiated by compounds that interfere with autophagosomal function. Combined treatment with bortezomib and autophagy inhibitors enhanced NOXA stability leading to super-induction of NOXA protein. In addition to established autophagy modulators, we identified the fatty acid synthase inhibitor orlistat to be an efficient autophagy inhibitor when used in combination with bortezomib. Accordingly, this combination synergistically induced apoptosis both in MCL cell lines and in patient samples.

**Conclusion:**

Our data demonstrate that CDK4 activity in MCL is critical for NOXA stabilization upon treatment with UPS inhibitors allowing preferential induction of cell death in cyclin D transformed cells. Under UPS blocked conditions, autophagy appears as the critical regulator of NOXA induction. Therefore, inhibitors of autophagy are promising candidates to increase the activity of proteasome inhibitors in MCL.

**Electronic supplementary material:**

The online version of this article (10.1186/s13045-018-0657-6) contains supplementary material, which is available to authorized users.

## Background

Mantle cell lymphoma (MCL) is a rare B cell neoplasia often characterized by an aggressive clinical course, relatively short response to conventional chemotherapy and frequent relapses [1]. The initial molecular pathogenic event of this malignancy is the t(11;14)(q13;q32) translocation leading to juxtaposition of the *CCND1* gene (coding for cyclin D1) to the immunoglobulin heavy chain complex (IgH) gene [2]. Normally, the G1 phase regulator cyclin D1 is not expressed at high levels in normal B cells but gets overexpressed as a result of this translocation [3]. As a binding partner and activator of the cyclin-dependent kinases CDK4 and CDK6, cyclin D1 plays a crucial role in G1-S transition and promotes proliferation by phosphorylating RB1 and preventing its inhibitory interaction with the E2Fs [4]. As CDK6 is hardly expressed in MCL cells, cyclin D1 mainly exerts its functions via CDK4 [5]. In addition to its role in cell cycle progression, cyclin D1 affects different cellular processes via both CDK4-dependent and CDK4-independent mechanisms [6]. Cyclin D1/CDK4 activity has also been shown to suppress the autophagic degradation machinery [7–11].

The proteasome inhibitor bortezomib which was approved for treatment of relapsed or refractory MCL was recently included in the first-line treatment of MCL [12, 13]. As there are still many patients who are poorly responding to bortezomib treatment, there is urgent need to elucidate the basis for bortezomib sensitivity and resistance [12]. Different resistance mechanisms to bortezomib have been proposed including accumulation of Bcl-2 protein in lymphoma cells, mutation as well as overexpression of proteasomal subunits in THP1 cells and plasmacytic differentiation, elevated nuclear factor-κB activity, or increased autophagy in MCL cells [14–18]. Combination of bortezomib with substances that target the DNA methyltransferase, histone acetylation, prosurvival chaperones, PI3K/AKT signaling, or the anti-apoptotic protein MCL1 have been shown to enhance the efficacy of bortezomib in MCL [19–23]. Work from our group revealed that extensive proteasomal degradation of the pro-apoptotic Bcl-2 family protein NOXA and corresponding low protein levels is crucial for sensitivity of MCL to the proteasome inhibitor bortezomib [24].

The aim of the present work is to investigate how cyclin D1 overexpression and aberrant CDK4 activity affect treatment efficacy of bortezomib in MCL. Furthermore, we seek to elucidate underlying molecular mechanisms and identify novel combinations to enhance bortezomib treatment in MCL.

## Methods

### Cell culture

The MCL cell lines Mino, Jeko-1, Rec-1, Jvm2, and Granta-519 were obtained from the German Collection of Microorganisms and Cell Cultures GmbH (Germany). NIH3T3 cells stably transfected with human CD40 ligand were a kind gift from Dr. Martina Seiffert from the German Cancer Research Center, DKFZ (Germany). All cell lines were tested for mycoplasma contamination and recently authenticated by short tandem repeat profiling. Cell lines as well as the primary MCL cells were cultivated in RPMI-1640 (Biochrom, Germany) supplemented with 20% fetal calf serum, 0.1 g/l penicillin-streptomycin (Gibco, Germany), and 2 mM L-glutamine (Biochrom, Germany).

### Reagents

Cycloheximide, 3-methyladenine, hydroxychloroquine, Spautin-1, orlistat, BEZ235, Cerulenin, and hydrogen peroxide were purchased from Sigma-Aldrich Chemie GmbH (Germany). Liensinine was obtained from ChemFaces (China) and bortezomib, carfilzomib, and palbociclib from Selleck Chemicals (USA).

### Protein expression analysis

Cells were lysed, sonicated, and boiled to obtain cellular proteins according to standard protocols. Western blots were performed using a SDS-PAGE Gel Electrophoresis system. Proteins were blotted on Amersham Protran 0.1 NC nitrocellulose membranes, except for LC3 which was blotted on Amersham Hybond P 0.2 PVDF membranes (GE Healthcare, USA). The primary antibodies used were anti-NOXA antibody (Calbiochem, USA), anti-cyclin D1 antibody (Santa Cruz Biotechnology, USA), anti-MCL1, RB1, β-actin, α-tubulin, ATG5, ATG7, LC3, CDK4, PUMA, BAX, BAK, and GAPDH (Cell Signaling, USA).

### Autophagolysosome detection

After treatment, samples containing 5 × 10^5^ to 1 × 10^6^ cells were stained with Cyto-ID Green Detection Reagent (Enzo Life Sciences, USA) according to manufacturer’s instructions for 30 min at 37 °C in the dark. Afterwards, cells were stained with AnnexinV-APC (BD Pharmingen, Germany) and subsequently analyzed by flow cytometry. The Cyto-ID fluorescence intensity of AnnexinV-APC negative cells was detected and compared by histogram overlays for the different treatments.

### mRNA expression analysis

RNA was extracted using RNeasy Mini Kit (QIAGEN N.V., Netherlands) and transcribed to cDNA according to standard protocols. *NOXA* (*PMAIP1*) expression was analyzed using TaqMan Gene Expression Assay Hs00560402_m1 (Applied Biosystems, USA) on 7900 HT Fast Real-Time PCR System (Applied Biosystems) according to the manufacturer’s instructions. TBP (Hs00427620_m1) was used for normalization of *NOXA* mRNA expression.

### Gene silencing

For gene silencing, we used siGENOME siRNA Reagents - Human SMARTpool siRNA (Dharmacon, UK). Sequences targeted by the siRNAs are shown in Additional file 1: Table S1. As control, siRNA (cosi) non-targeting siRNA#1 (Dharmacon, UK) was used. For electroporation, the Nucleofector™ II/2B, the Cell Line Nucleofector® Kit V, and the program X-001 (Lonza Group Ltd., Basel, Switzerland) were used. Knockdown efficacy was analyzed by Western blot.

### Cell death detection

Cell death was assessed by staining the cells with AnnexinV-FITC (BD Pharmingen, Heidelberg, Germany) and propidium iodide (PI) (Sigma-Aldrich, Steinheim, Germany) and subsequent analysis by flow cytometry. Combination index (CI) values were determined with the CalcuSyn Software (Biosoft, UK), whose algorithm is based on the Chou and Talalay’s method [25]. A combination index smaller than 0.9 indicates a synergistic effect between two substances.

### Measurement of cell cycle distribution

Analysis of cell cycle distribution was carried out using BrdU staining as previously described [26].

### Determination of protein half-life

The half-life of NOXA protein was determined by treating the cells with 20 μg/ml cycloheximide and harvesting cell pellets at different time points for subsequent analysis by Western blot.

### Statistics

Data are obtained from at least three independent experiments and expressed as standard deviation (SD) of the mean. Statistics were calculated using GraphPadPrism 4.0 software (GraphPad Software, La Jolla, CA, USA). Changes in paired samples were analyzed using two-sided paired *t* test, and results were considered statistically significant when *p* < 0.05 (*p < 0.05; **p < 0.01; ***p < 0.001).

## Results

### Cyclin D1-driven CDK4 activity is required for cell death and induction of NOXA protein upon proteasome inhibitor treatment

**Fig. 1:**
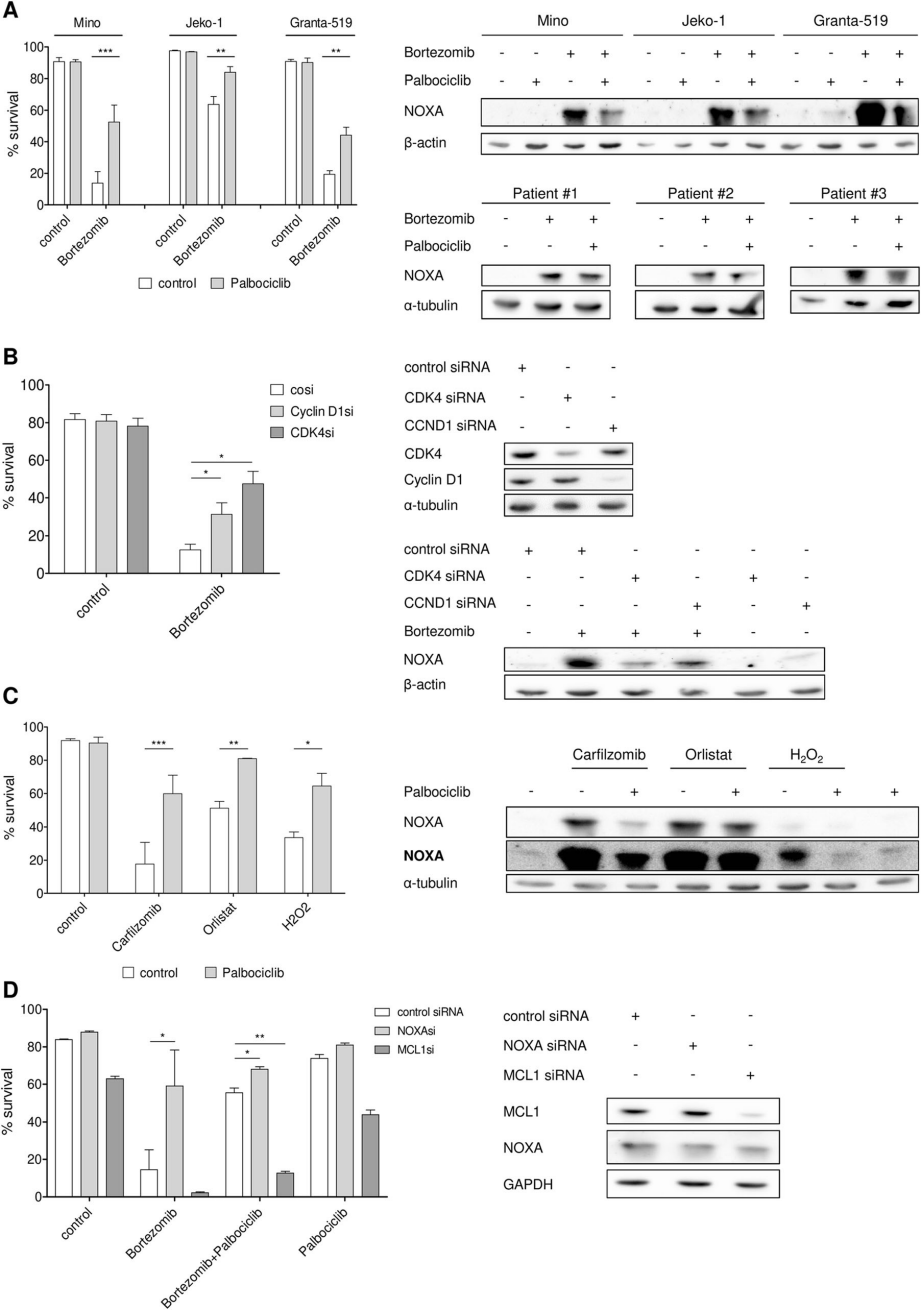
Cyclin D1-driven CDK4 activity is required for cell death induction of NOXA accumulating agents. **a** CDK4 inhibition by palbociclib antagonizes bortezomib-induced cell death and NOXA accumulation. MCL cell lines Jeko-1 and Granta-519 were treated with 300 nM and Mino with 100 nM palbociclib for 16 h and subsequently co-treated with 8 nM bortezomib for 24 h. Primary MCL cells were exposed to 50 ng/ml interleukin-10, 50 ng/ml B cell activating factor, 1 ng/ml of insulin-like growth factor-1, 1 ng/ml interleukin-6, and co-cultured with the CD40 ligand-expressing cell line 3T3. After 8 h of co-culture, cells were treated with 300 nM palbociclib for 16 h and subsequently co-treated with 10 nM bortezomib. The 3T3 cell line was irradiated with 30 Gy 1 day before co-culture. After 8 h, protein expression was analyzed (right), and after 24 h, cell death was assessed by AnnexinV-PI staining (left). **b** Knockdown of cyclin D1 and CDK4 antagonizes bortezomib-induced cell death and NOXA accumulation. MCL cell line Mino was transfected with siRNA targeting *CCND1* and *CDK4*. Twenty-four hours after transfection, cells were treated with 8 nM bortezomib. After 8 h, protein expression was analyzed (right) and cell death was assessed by AnnexinV-PI staining (left) 24 h post-treatment. **c** CDK4 inhibition antagonizes cell death of NOXA inducing substances and NOXA accumulation. MCL cell line Mino was treated with 100 nM palbociclib for 16 h and subsequently co-treated with either 8 nM carfilzomib, 20 μM orlistat, or 500 μM hydrogen peroxide. After 8 h, protein expression was analyzed (right), and after 24 h, treatment cell death was assessed by AnnexinV-PI staining (left). **d** MCL cell line Mino was transfected with siRNA targeting *PMAIP1* and *MCL1* and treated with 100 nM palbociclib. Twenty-four hours after transfection, protein expression was analyzed (right) and cells were treated with 8 nM bortezomib. Cell death was assessed by AnnexinV-PI staining 24 h post-treatment (left). Data represent means ± S.D. from three independent experiments

Sensitivity of MCL cells to inhibitors of the ubiquitin-proteasome pathway (UPS), such as bortezomib, has been associated with the BH3 only protein NOXA [24, 27, 28]. A recently published preclinical study also demonstrated a link between cyclin D1 expression and bortezomib sensitivity in multiple myeloma cell lines [29]. However, it is not well established if bortezomib sensitivity is dependent on the kinase activity of the cyclin D1/CDK4 complex and if NOXA is affected by this activity in MCL cells. To address this issue, we first incubated MCL cell lines Mino, Jeko-1, and Granta-519 with or without palbociclib, a potent and selective CDK4/6 inhibitor and co-treated the cell lines with bortezomib. In all three MCL cell lines investigated, co-treatment with palbociclib partially rescued cells from bortezomib-induced cell death (Fig. 1a, left). Co-treatment of bortezomib with palbociclib led to a reduced induction of NOXA protein in the MCL cell lines and in patient-derived primary MCL cells (Fig. 1a, right). Importantly, palbociclib treatment inhibited CDK4-dependent phosphorylation of RB1 in Mino, Jeko-1, and Granta-519 (Additional file 2: Figure S1). In order to prevent excessive cell death caused by the extended cultivation duration during the co-treatment schedule, we co-cultivated the primary MCL cells with CD40 ligand-expressing fibroblasts and different cytokines. This resulted in an acquired therapy resistance of the primary MCL cells which did not exhibit cell death after bortezomib treatment with or without palbociclib co-treatment (data not shown). The development of therapy resistance mechanisms after co-culturing primary MCL cells is known and involves the activation of survival pathways and the increased expression of anti-apoptotic Bcl-2 family proteins [30, 31]. In order to analyze if the antagonism on the bortezomib effect after palbociclib treatment was dependent on inhibition of cyclin D1-driven CDK4 activity, we performed siRNA-mediated knockdown of either cyclin D1 or CDK4. We observed a comparable reduction in bortezomib sensitivity in MCL cell lines Mino and Jeko-1 after knockdown of cyclin D1 or CDK4 (Fig. 1b, left and Additional file 3: Figure S2). In addition, NOXA induction after bortezomib treatment was also antagonized by knockdown of cyclin D1 or CDK4 (Fig. 1b, right). Double knockdown of cyclin D1 and CDK4 did not increase the antagonism on bortezomib-induced cell death (Additional file 4: Figure S3), indicating that in this context, both of these proteins exert their function through the same pathways.

Collectively, these data demonstrate that cyclin D1/CDK4 activity is required for effective accumulation of NOXA protein and thus also for complete execution of bortezomib-induced cell death in MCL.

We next asked if inhibition of CDK4 activity might also reduce sensitivity to other clinically relevant proteasome inhibitors such as carfilzomib. In contrast to bortezomib, which not only reversibly blocks the chymotrypsin- and caspase-like activity of the proteasome but also binds different serine proteases, carfilzomib is more specific and irreversibly binds the N-terminal threonine active sites of the proteasome [32, 33]. As shown in Fig. 1c, the effect of palbociclib on carfilzomib-induced cell death and NOXA accumulation in MCL cell line Mino was identical to that observed with bortezomib. Importantly, this could also be shown for other compounds which have been identified to be effective in MCL cells via upregulation of NOXA, such as the fatty acid synthase inhibitor orlistat (Fig. 1c; [24]) as well as for hydrogen peroxide (Fig. 1c; [34]). Similar results were also observed in the MCL cell line Jeko-1 (Additional file 5: Figure S4). In order to confirm that the inability to induce cell death after co-treatment of bortezomib with palbociclib is dependent on NOXA, we performed knockdowns of NOXA and its anti-apoptotic binding partner MCL1. NOXA knockdown rescued bortezomib-induced cell death (Fig. 1d). Furthermore, knockdown of MCL1 reversed the palbociclib-mediated antagonism on bortezomib-induced cell death, whereas NOXA knockdown further increased the antagonism on cell death induction (Fig. 1d).

Together, these observations indicate that cyclin D1-driven CDK4 activity is required for effective upregulation of NOXA and concomitant induction of cell death not only upon treatment with proteasome inhibitors but also other NOXA protein inducing agents.

### Inhibition of CDK4 activity antagonizes proteasome inhibitor-mediated stabilization of NOXA by autophagy-driven proteolysis

Previous studies have shown that inhibition of the proteasome leads to induction of *NOXA* transcript [28, 35]. However, the predominant mechanism by which NOXA protein is enhanced seems to be protein stabilization by targeting the rapid NOXA protein turnover both in MCL and in melanoma cells [24, 36]. To uncover the mechanism by which CDK4 inhibition diminishes NOXA protein in bortezomib-treated cells, we first quantified *NOXA* RNA levels. We found only minor differences of *NOXA* transcript induction upon bortezomib in cells pre-incubated with or without palbociclib, indicating that transcriptional regulation of *NOXA* upon bortezomib is not the predominant effect of CDK4 inhibition on *NOXA* regulation (Additional file 6: Figure S5). Palbociclib antagonism on bortezomib-induced cell death might also be mediated by alterations in cell cycle distribution. We therefore performed a knockdown of RB1 to allow cells to progress to s-phase while CDK4 is inhibited. Even though cells progressed to s-phase (Additional file 7: Figure S6, left panel), palbociclib antagonized bortezomib-induced cell death in RB1 knockdown cells in the same extent as cells transfected with control siRNA (Additional file 7: Figure S6, middle panel). Furthermore, bortezomib treatment led to a similar decrease of s-phase cells as palbociclib treatment (Additional file 7: Figure S6, left panel). Consequently, we conclude that it is unlikely that cell cycle distribution does mediate palbociclib effects on bortezomib-induced cell death.

**Fig. 2:**
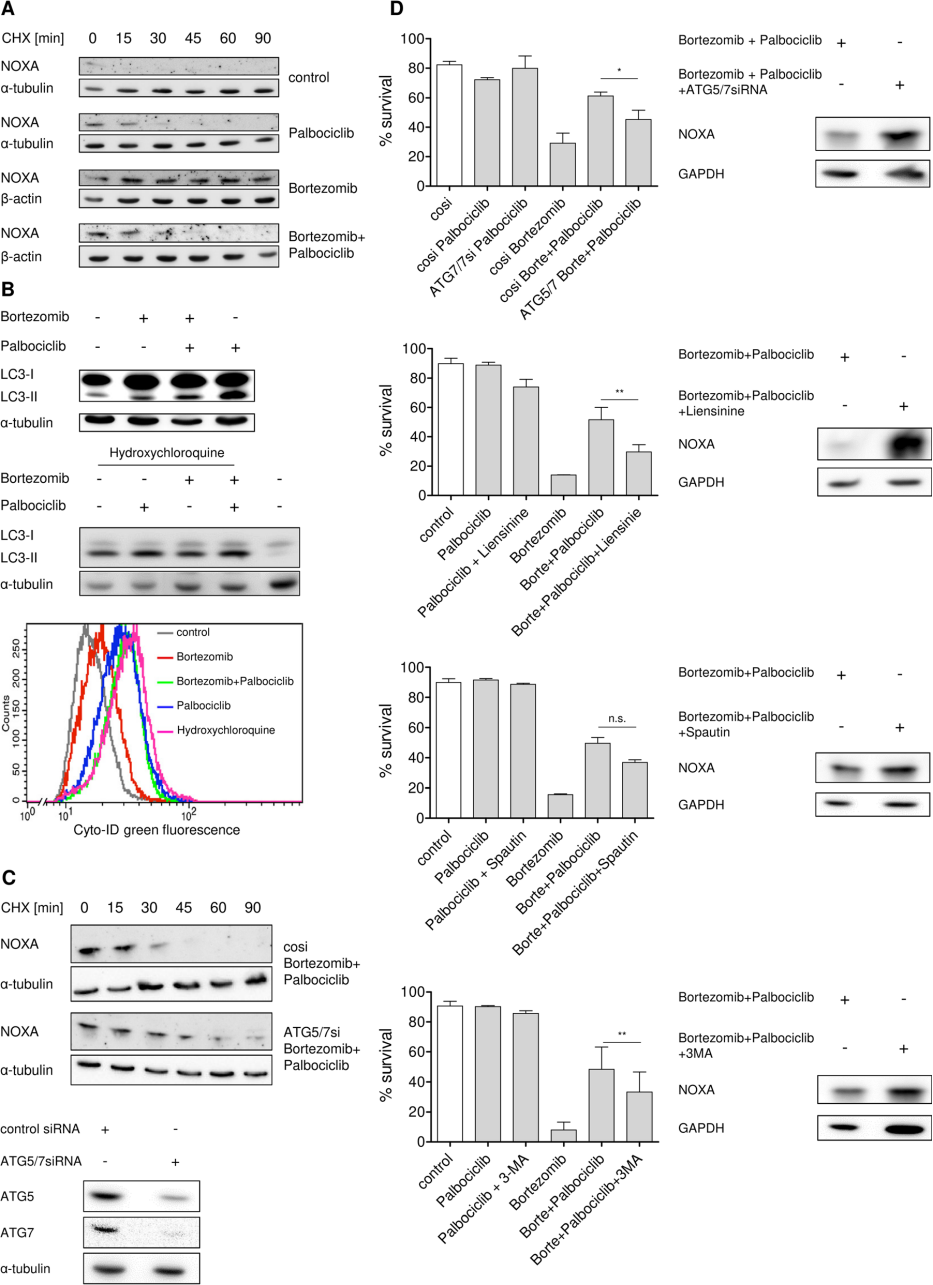
Proteasome inhibitor-mediated stabilization of NOXA is abrogated through an autophagy-driven proteolysis. **a** Increased half-life of Noxa protein after bortezomib treatment is diminished after palbociclib co-treatment. MCL cell line Mino was treated with 100 nM palbociclib for 16 h and subsequently co-treated with 8 nM bortezomib. After 8 h co-treatment, 20 μg/ml cycloheximide was added to the cells and samples were harvested 0, 15, 30, 45, 60, and 90 min after cycloheximide exposition for Western blot analysis. **b** Palbociclib treatment induces autophagy. MCL cell line Mino was treated with 100 nM palbociclib for 16 h and subsequently co-treated with 8 nM bortezomib. After 8 h, protein expression was analyzed (upper panel). MCL cell line Mino was treated with 40 μM hydroxychloroquine and 100 nM palbociclib for 16 h and subsequently co-treated with 8 nM bortezomib. After 8 h, protein expression was analyzed (middle panel). MCL cell line Mino was treated with 40 μM hydroxychloroquine for 24 h or treated with 100 nM palbociclib for 16 h and subsequently co-treated with 8 nM bortezomib for 24 h. After treatment, autophagic vesicles were measured by Cyto-ID staining (lower panel). **c** Knockdown of autophagy-related genes reverses the palbociclib-mediated antagonism on bortezomib-mediated stabilization of NOXA. MCL cell line Mino was transfected with siRNA targeting *ATG5* and *ATG7*. Twenty-four hours after transfection, cells were treated with 100 nM palbociclib for 16 h and subsequently co-treated with 8 nM bortezomib. After 8 h, cells were exposed to 20 μg/ml cycloheximide and samples were harvested 0, 15, 30, 45, 60, and 90 min after cycloheximide addition and analyzed by Western blot. **d** Knockdown of autophagy-related genes as well as autophagy inhibitors counteract palbociclib-mediated antagonism on bortezomib-induced cell death and NOXA induction. MCL cell line Mino was transfected with siRNA targeting *ATG5* and *ATG7*. Twenty-four hours after transfection, cells were treated with 100 nM palbociclib for 16 h and subsequently co-treated with 8 nM bortezomib. After 8 h, protein expression was analyzed (upper right) and cell death was assessed by AnnexinV-PI staining 24 h post-treatment (upper left). MCL cell line Mino was treated with 100 nM palbociclib and 20 μM liensinine (upper middle panel), 5 μM Spautin-1 (lower middle panel), or 2 mM 3-MA (lower panel) for 16 h and subsequently co-treated with 8 nM bortezomib. After 8 h, protein expression was analyzed (right) and cell death was assessed by AnnexinV-PI staining 24 h post-treatment (left). Data represent means ± S.D. from three independent experiments

We therefore studied NOXA protein stability using cycloheximide pulse-chase experiments. In accordance with previous studies [24, 37], bortezomib significantly prolonged NOXA half-life in MCL cell line Mino (Fig. 2a), whereas palbociclib alone had no effect on NOXA protein stability. Interestingly, the stabilizing effect of bortezomib was almost completely antagonized when co-treated with palbociclib indicating that CDK4 inhibition activates a proteasome-independent degradation of NOXA.

Several groups have shown that CDK4 activity [7–11] can suppress autophagy, which represents another central degradation mechanism delivering cytosolic proteins, aggregates, or organelles to lysosomes [38]. In line with this, we observed an increase of the autophagy marker LC3-II in MCL cell line Mino upon co-treatment of bortezomib with palbociclib or after treatment with palbociclib alone (Fig. 2b, upper panel). LC3-II protein levels can be elevated either because the completion of autophagy is impaired which leads to an accumulation of autophagosomes or because there is an actual increase of the autophagic flux [39]. To differentiate this, we treated the MCL cell line Mino with palbociclib and the downstream autophagy inhibitor hydroxychloroquine which inhibits the acidification of lysosomes, completely blocks autophagy in its final step and therefore leads to the accumulation of autophagosomes [40]. Hydroxychloroquine treatment induced LC3-II protein levels which, importantly, were further increased by co-treatment with palbociclib (Fig. 2b, middle panel). We can thereby conclude that inhibition of CDK4 activity results in increased autophagic flux. In addition, direct staining of autophagic vacuoles with the autophagy Cyto-ID Green dye and subsequent cytometric analysis corroborated our results that we obtained by analyzing LC3-II protein expression. CDK4 inhibition with palbociclib alone or after co-treatment with bortezomib induced an increase in green fluorescence, indicative of elevated levels of autophagic vesicles (Fig. 2b, lower panel). Similar results were observed in MCL cell line Jeko-1, where palbociclib treatment led to an increase in green fluorescence (Additional file 8: Figure S7A). Furthermore, co-treatment of palbociclib with hydroxychloroquine showed higher green fluorescence than hydroxychloroquine alone (Additional file 8: Figure S7A), corroborating our result that CDK4 inhibition results in increased autophagic flux (Fig. 2b, middle panel). Short-term treatment of MCL cell line Mino with Palbociclib, however, did not led to an increase in autophagic vacuoles (Additional file 8: Figure S7B), indicating that it requires more time to activate intermediate signals that induce autophagy.

To test whether the antagonizing effect of palbociclib on bortezomib-induced NOXA stability is mediated by activating an autophagy-lysosome pathway (ALP)-dependent degradation of NOXA, we performed RNAi-mediated knockdowns of autophagy-related proteins (ATG5/7) and investigated NOXA protein stability. Indeed, compared to control siRNA genetic blockade of autophagy by pre-incubation with *ATG5/7* siRNA prolonged NOXA half-life in the MCL cell line Mino co-treated with palbociclib (Fig. 2c), reaching a protein stability comparable to that observed in cells treated with bortezomib alone (Fig. 2a). Importantly, in cells co-treated with palbociclib, genetic or pharmacologic inhibition of autophagy with Spautin-1, liensinine, or 3-methyladenine (3-MA) also restored the effect of bortezomib on cell death induction in MCL cell lines Mino and Jeko-1 (Fig. 2d, left and Additional file 9: Figure S8) and on NOXA induction in MCL cell line Mino (Fig. 2d, right). These results indicate that inhibition of CDK4 antagonizes bortezomib efficacy via an autophagy-mediated degradation of NOXA.

### Inhibition of autophagy synergizes with bortezomib-induced cell death and NOXA stabilization in MCL

**Fig. 3:**
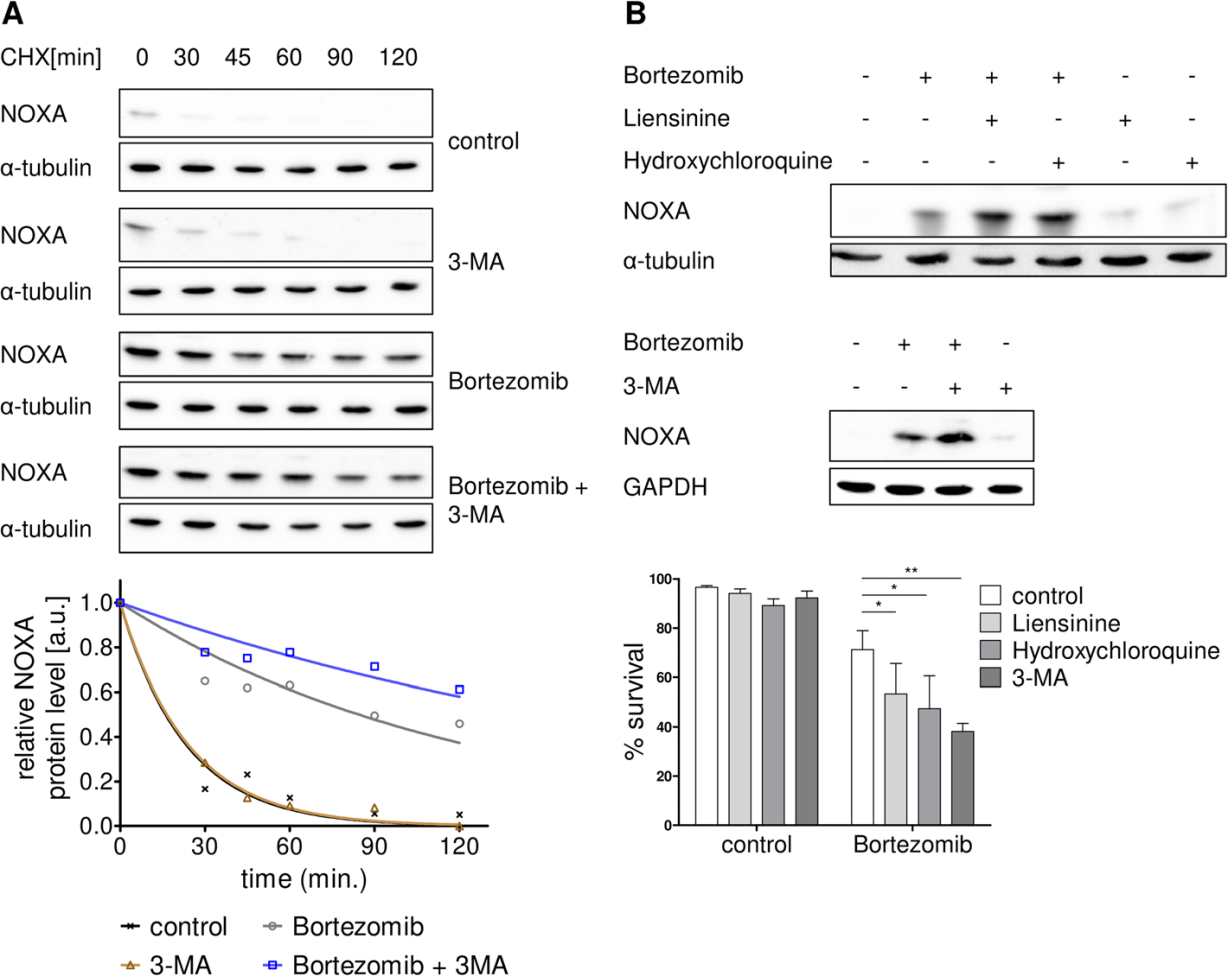
Autophagy inhibitors potentiate bortezomib-induced cell death through NOXA stabilization. **a** Co-treatment of bortezomib with an autophagy inhibitor potentiates half-life increase of NOXA protein. MCL cell line Jeko-1 was treated with 2 mM 3-MA for 16 h and subsequently co-treated with 7 nM bortezomib. After 14 h, 20 μg/ml cycloheximide was added to the cells and samples were harvested 0, 30, 45, 60, 90, and 120 min after cycloheximide exposition for Western blot analysis (upper panel) and corresponding densitometric analysis of NOXA protein stability (lower panel). **b** Co-treatment of Bortezomib with autophagy inhibitors potentiates cell death induction as well as NOXA protein accumulation. MCL cell line Jeko-1 was treated with 20 μM liensinine, 40 μM hydroxychloroquine, or 2 mM 3-MA for 16 h and subsequently co-treated with 8 nM bortezomib. After 8 h, protein expression was analyzed (upper panel), and after 24 h treatment, cell death was assessed by AnnexinV-PI staining (lower panel). Data represent means ± S.D. from three independent experiments

If NOXA stability is targeted by UPS and ALP-mediated proteolysis, we would expect an enhanced half-life of NOXA upon inhibition of both the proteasome and the autophagy pathway as well as an increase of cell death as compared to proteasome inhibition alone. To address this issue, we used a cell line with reduced sensitivity to proteasome inhibitors, namely Jeko-1 (Fig. 1), and performed cycloheximide pulse-chase experiments. For evaluation of NOXA protein stability, cells were incubated in presence or absence of the UPS inhibitor bortezomib, the autophagy inhibitor 3-MA, and the combination of both. Interestingly, inhibition of autophagy alone had no effect on NOXA protein half-life (Fig. 3a) indicating that ALP has a minor role for NOXA degradation in cells with active UPS. However, compared to inhibition of the proteasome alone, combined inhibition of the ubiquitin-proteasome and the autophagy-lysosome pathways led to a significant increase of NOXA stability as shown by Western blot analysis (Fig. 3a, upper panel) and corresponding densitometric quantification (Fig. 3a, lower panel). Importantly, this elevated NOXA accumulation upon combined inhibition of autophagy and proteasome was accompanied by a significantly enhanced cell death as compared to proteasome inhibition alone (Fig. 3b, lower panel). Analogous to the results obtained with 3-MA, combination of bortezomib with the autophagy inhibitors liensinine or hydroxychloroquine also resulted in an elevated NOXA accumulation as well as enhanced cell death compared to proteasome inhibition alone (Fig. 3b). In line with this, autophagy inhibitors also potentiated bortezomib-induced cell death in another bortezomib-resistant cell line, namely Rec-1 (Additional file 10: Figure S9).

Together, these results indicate that bortezomib-mediated accumulation of NOXA is partially hampered by a parallel degradation of NOXA via autophagy. Therefore, combined inhibition of both UPS and autophagy potentiates both stabilization of NOXA protein and induction of cell death in MCL cells.

### Orlistat acts synergistically with bortezomib to accumulate NOXA and induce potent cell death in MCL cells

Previous work of our group showed that MCL cells can be efficiently treated using the fatty acid synthase inhibitor (FASNi) orlistat [24, 41]. This cell death relies on the efficient stabilization of NOXA protein. The FASNi orlistat has been shown to block palmitate synthesis [42]. Interestingly, many studies have shown that palmitate levels are linked to ER stress and autophagy regulation [43–45]. Therefore, we investigated if bortezomib effects could be potentiated by orlistat co-treatment in analogy to the observation with autophagy inhibitors (Fig. 3).

**Fig. 4:**
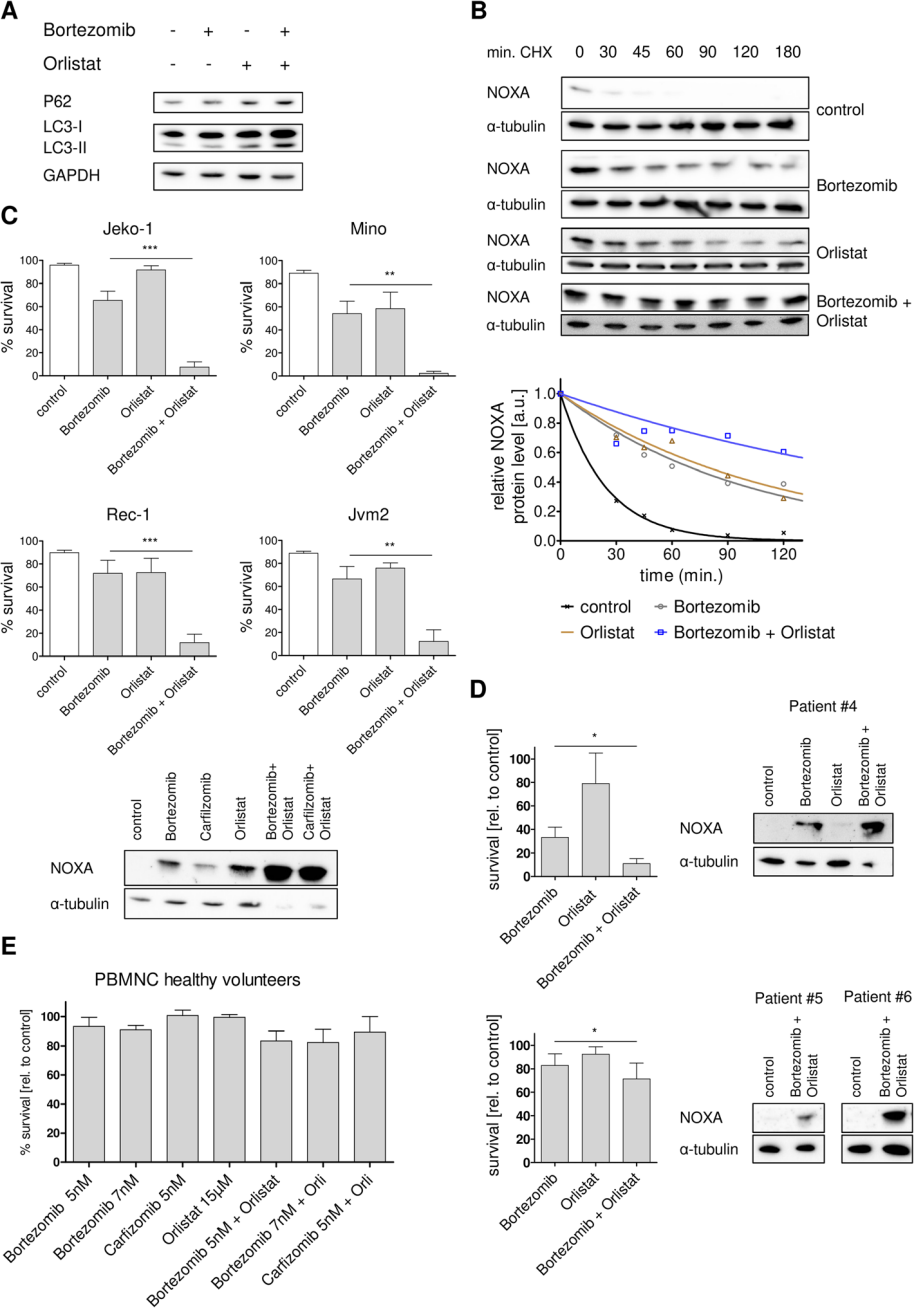
FASNi modulates autophagy upon proteasome inhibition and leads to synergistic cell death in MCL cells. **a** Co-treatment of bortezomib with the fatty acid synthase inhibitor orlistat blocks autophagy. MCL cell line Mino was treated with 15 μM orlistat and 7 nM bortezomib. After 8 h, samples were harvested for Western blot analysis. **b** Inhibition of proteasome and fatty acid synthase further increases NOXA half-life. MCL cell line Jeko-1 was treated with 15 μM orlistat and 7 nM bortezomib. After 14 h, 20 μg/ml cycloheximide was added to the cells and samples were harvested 0, 30, 45, 60, 90, 120, and 180 min after cycloheximide exposition for Western blot analysis (upper panel) and quantification of NOXA protein stability (lower panel). **c** Co-treatment of bortezomib with orlistat induces cell death in MCL cell lines. MCL cell lines were treated with 15 μM orlistat and 7 nM (Jeko-1 and Rec-1) or 5 nM (Mino and Jvm2) bortezomib. After 14 h, protein expression was analyzed (Jeko-1), and after 24 h, cell death was assessed by AnnexinV-PI staining. **d** Co-treatment of bortezomib with orlistat induces cell death in primary MCL cells. Primary MCL cells were treated with 15 μM orlistat and 7 nM (four patients, upper panel) or 5 nM (six patients, lower panel) bortezomib. After 24 h, samples were taken for Western blot analysis (left) and cell death was assessed by AnnexinV-PI staining (right). **e** Healthy lymphocytes and monocytes are hardly affected by co-treatment of proteasome inhibitors with orlistat. Peripheral blood mononuclear cells were treated with 15 μM orlistat and 5 nM or 7 nM bortezomib. After 24 h, cell death was assessed by AnnexinV-PI staining. Data represent means ± S.D. from three independent experiments

Co-treatment of bortezomib with the fatty acid inhibitor orlistat led to a blockade of autophagic degradation in MCL cell line Mino (Fig. 4a), which is characterized by an elevated LC-3 II protein expression and a concomitant accumulation of the P62 protein which is exclusively degraded by autophagy [46]. Consequentially, orlistat prolonged bortezomib-mediated NOXA half-life in bortezomib-resistant cell line Jeko-1 as shown by Western blot analysis (Fig. 4b, upper panel) and corresponding densitometric quantification (Fig. 4b, lower panel). This enhanced protein stability resulted in super-induction of NOXA protein and an almost complete loss of viability in four MCL cell lines characterized by different sensitivity to bortezomib monotherapy (Fig. 4c). The same results were obtained with carfilzomib (Additional file 11: Figure S10) indicating that the potentiating effect was not due to a differential bortezomib uptake or substance cross-interaction.

Changes in expression levels of other apoptotic proteins apart from NOXA after combination treatments were not observed (Additional file 12: Figure S11). Treatment of the MCL cell line Jeko-1 with different combinations of orlistat and bortezomib or carfilzomib resulted in synergistic cell death even at low concentrations of bortezomib and carfilzomib (Additional file 13: Figure S12). Cell death induced by these combinations was dependent on caspase activity and could be blocked by pre-incubation with the pan-caspase inhibitor Z-VAD-FMK (Additional file 11: Figure S10). Importantly, similar effects were also observed in patient-derived primary MCL cells. Combination of orlistat together with bortezomib led to an enhanced NOXA protein expression and significantly potentiated the cytotoxic effect of bortezomib alone (when applied at 7 nM; Fig. 4d upper panel). Even a low bortezomib concentration of 5 nM which had only minor effects in monotherapy led to a significant increase of cell death when combined with orlistat (Fig. 4d, lower panel). Of note, this combinatory treatment had only minor effects on peripheral blood mononuclear cells (PBMNC) from healthy donors (Fig. 4e).

Collectively, these data demonstrate that the combination of bortezomib with a FASNi provides a potent tool to effectively induce MCL cell death via super-induction of NOXA presumably by parallel inhibition of the proteasome as well as the autophagic degradation machinery.

### Effective stabilization of NOXA and concomitant induction of apoptosis in MCL cells requires cyclin D1-CDK4 activity

**Fig. 5:**
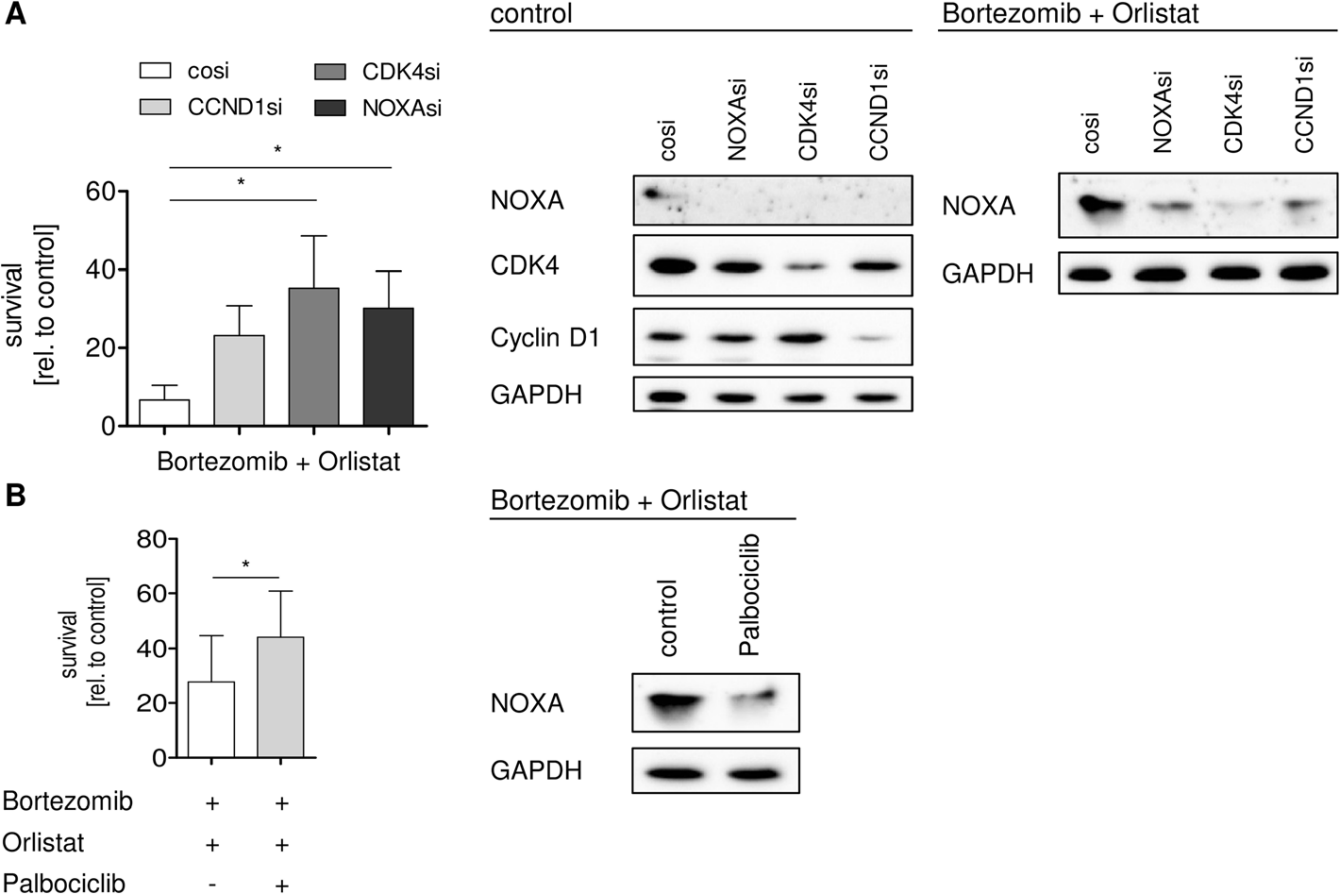
CDK4 activity is required for cell death induction after combined fatty acid synthase and proteasome inhibition. **a** Knockdown of either cyclin D1 or CDK4 antagonizes cell death as well as NOXA protein induction as efficient as knockdown of NOXA. MCL cell line Jeko-1 was transfected with siRNA targeting *NOXA*, *CCND1*, and *CDK4*. Twenty-four hours after transfection, cells were treated with 15 μM orlistat and 7 nM bortezomib. After 14 h, protein expression was analyzed (right) and cell death was assessed by AnnexinV-PI staining (left) 24 h post-treatment. **b** Co-treatment of bortezomib with orlistat is antagonized by palbociclib treatment. MCL cell line Jeko-1 was treated with 300 nM palbociclib and subsequently co-treated with 15 μM orlistat and 7 nM bortezomib. After 14 h, protein expression was analyzed (right) and cell death was assessed by AnnexinV-PI staining (left). Data represent means ± S.D. from three independent experiments

After demonstrating that cyclin D1-CDK4 activity is important for response to bortezomib in MCL cells (Fig. 1), we next asked if this also holds true for the more effective combination of bortezomib with orlistat. Indeed, knockdown of cyclin D1 or CDK4 in MCL cell line Jeko-1 blocked accumulation of NOXA upon combinatory treatment as effective as direct NOXA knockdown (Fig. 5a). Analogous to bortezomib alone, pharmacological inhibition of CDK4 by palbociclib prior to combination treatment was sufficient to abrogate NOXA accumulation (Fig. 5b). Again, consistent with the effects observed in cells treated with bortezomib alone, genetic or pharmacologic blockade of cyclin D1/CDK4 also partially rescued the more effective apoptosis upon co-treatment with orlistat.

Together, these results demonstrate that effective NOXA accumulation and induction of cell death after combined proteasome and fatty acid synthase inhibition is dependent on cyclin D1/CDK4 activity in MCL cells.

## Discussion

In MCL stabilization of the short-lived pro-apoptotic BH3-only protein, NOXA is a major determinant for its sensitivity to bortezomib and other therapeutics [24, 28]. Our results show that the aberrant cyclin D1/CDK4 activity in MCL is critical for NOXA protein induction and thereby directly contributes to cell death triggered by proteasome inhibition. Similar to MCL, in multiple myeloma, cyclin D1 overexpression is also frequently observed and the disease is responding well to proteasomal inhibition [47, 48]. Interestingly, the susceptibility to bortezomib was also shown to be dependent both on cyclin D1 and on NOXA protein expression [29, 49]. In addition, a correlation between cyclin D1 overexpression and response to bortezomib treatment was also shown in breast cancer [50]. We show that in MCL, both induction of NOXA protein and cell death by bortezomib are largely abolished when CDK4 activity is blocked by pharmacologic inhibition or genetic manipulation. Furthermore, our experiments clarify that autophagy is the biological mechanism underlying the interaction between proteasome inhibition and CDK4 activity in the regulation of cell death in MCL cells. When MCL cells are exposed to bortezomib, NOXA can only be efficiently induced if the aberrant cyclin D1/CDK4 activity inhibits autophagy. This allows the very efficient induction of NOXA protein and explains the preferential sensitivity of cyclin D1 transformed cells to bortezomib.

The autophagy-lysosomal degradation pathway is known to be suppressed by cyclin D1/CDK4 activity [8–10]. The exact mechanism of how autophagy and CDK4 activity is linked in MCL remains to be determined. It has recently been reported that autophagy triggered by CDK4 inhibition relies on the induction of reactive oxygen species (ROS) [9]. Other reports identified AMPK signaling as a link between CDK4 activity and autophagy, as AMPK can directly phosphorylate the autophagy activating kinase ULK1 or inhibit the mTOR pathway which again blocks autophagy by inhibiting ULK1 [51–53]. Of note, the mTOR inhibitor Everolimus has been shown to induce autophagy in MCL [54]. In line with these data, we also observed an antagonism on bortezomib-induced cell death by the dual PI3K/mTOR inhibitor BEZ235 as well as by the AMPK activator metformin (data not shown).

We show for the first time that in the absence of proteasomal activity, NOXA can be degraded by the autophagy-lysosome pathway. Many proteins are degraded by both autophagosome and the proteasome [55]. Proteasomal degradation of NOXA has been extensively studied [56]. It is known that proteasome inhibition may reveal a crosstalk between the ubiquitin-proteasome system and the autophagy-lysosomal pathway enabling a so-called compensatory autophagy [57]. Whether NOXA can be directed to the autophagosomal degradation machinery has never been described. Chaperone-mediated autophagy (CMA) is a selective catabolic pathway that mediates proteins to lysosomes. This pathway involves specific chaperones, the lysosome-associated membrane protein type 2A (LAMP-2A) and the presence of a peptide motif biochemically related to KFERQ [58]. As this motif is mandatory for CMA and as it is not present in the NOXA protein sequence, it is unlikely that NOXA is a target for CMA. Macroautophagy (generally referred to as autophagy), however, involves proteins of the ATG8 family which promote the entry of cargo receptors into the autophagy pathway through interaction with the LC3-interacting regions (LIR) [59]. Interestingly, a LIR motif can be found in the NOXA protein sequence (Additional file 14: Figure S13) and might therefore target NOXA for ALP-dependent proteolysis.

Our findings have important implications for further development of combinatorial strategies with proteasomal inhibitors. Co-treatment of bortezomib with agents that either induce autophagy or abrogate its inhibition such as palbociclib reduces the efficacy of proteasome inhibitors. In contrast, combined treatment with agents that inhibit autophagy and/or lysosomal degradation might act synergistically with proteasome inhibitors. The clinical consequences are therefore twofold: When combining proteasome inhibitors with other chemotherapeutics, particular care must be taken. Co-treatment with a chemotherapeutic agent might cause nutrient starvation, inhibit the PI3K/AKT/mTOR pathway, activate AMPK signaling pathway, and induce ROS or ER stress which could eventually lead to prosurvival autophagy induction. Furthermore, co-treatment could impair CDK4 activity which, as we have shown, prevents prosurvival autophagy upon proteasome inhibition in MCL. On the other hand, proteasome inhibitors can be combined with approved therapeutics that block autophagy. Chloroquine and its derivative hydroxychloroquine, which have been initially FDA approved as antimalarial drug, are currently repurposed to inhibit autophagy and are well tolerated in clinical use [60]. Interestingly, a phase 1 clinical trial including patients with relapsed/refractory myeloma showed promising results for the combination of bortezomib with the autophagy inhibitor hydroxychloroquine corroborating our results [61]. More importantly, there are several more potent and specific autophagy inhibitors under preclinical investigation [62].

As we have shown here with the fatty acid synthase inhibitor orlistat, it is a highly interesting approach to investigate established compounds for their potential to regulate autophagy. This approach of re-positioning conventional drugs to target autophagy and induce cancer cell death was already shown for several therapeutics [63]. Orlistat might impair the autophagic machinery due to a reduction in palmitate levels [43–45]. Our group has previously shown that orlistat-induced cell death in MCL is indeed palmitate-dependent [24]. Alternatively, there are recent reports showing that lipid droplets and their components triglycerides are linked to endoplasmic reticulum homeostasis and autophagy regulation [64]. In line with this, another fatty acid synthase inhibitor Cerulenin has also been shown to inhibit autophagy due to lipid droplet downregulation [65]. Furthermore, we observed that co-treatment of bortezomib with Cerulenin potentiates cell death in a similar manner as orlistat does (data not shown).

NOXA contains six lysine sites and is substrate for polyubiquitination [56]. The only reported polyubiquitin chains for NOXA are K11 or K48 linkages [36, 66]. Although the K63 linkage has been shown to direct proteins preferentially towards autophagy instead of proteasomal degradation, all ubiquitin linkages including K11 and K48 have been shown to be involved in autophagosomal trafficking [67, 68]. Interestingly, our results show that orlistat is more effective in potentiating proteasomal inhibitor-mediated cell death and NOXA accumulation as compared to autophagy inhibitors. Our group previously demonstrated that orlistat interferes with NOXA ubiquitination [24]. Polyubiquitination is important for substrate selectivity of UPS as well as ALP [57, 69]. Therefore, in addition to orlistat blocking the late steps of ALP process (Fig. 4a), orlistat potentially impairs proteasomal degradation as well as trafficking of NOXA protein to autophagy in parallel and thus subsequently causing hyper-accumulation of NOXA protein.

In addition to the aforementioned mechanisms, many chemotherapeutics have shown to cause oxidative stress [70]. Therefore, it might be a promising approach investigating concomitant autophagy induction as oxidative stress regulates autophagy [71]. There is also growing insight into the interaction between DNA damage response and autophagy regulation [72]. It will be highly interesting to investigate if standard therapy regimes like R-CHOP could benefit from autophagy inhibitors.

## Conclusion

Our data demonstrate for the first time that the high CDK4 activity in MCL is a prerequisite for the response to bortezomib therapy as well as for other compounds that require stabilization of NOXA for efficient cell death induction. We found out that the underlying molecular mechanism is a mitigated autophagy due to high CDK4 activity, offering novel combination treatment strategies in MCL and bortezomib-resistant cells. We show that NOXA can be degraded by the autophagosome and that blocking the residual autophagic activity combined with proteasome inhibition leads to highly efficient cell death in MCL due to super induction of NOXA protein.

## Additional files

Additional file 1: **Table S1.** Sequences targeted by the siRNAs used for gene silencing. (TIFF 883 kb)

Additional file 2: **Figure S1.** CDK4 inhibition by palbociclib treatment inhibits RB1 phosphorylation. MCL cell lines Jeko-1 and Granta-519 were treated with 300 nM and MCL cell line Mino with 100 nM palbociclib. After 16 h, proteins were analyzed by Western blot. (TIFF 360 kb)

Additional file 3: **Figure S2.** Knockdown of cyclin D1 and CDK4 antagonizes bortezomib-induced cell death. MCL cell line Jeko-1 was transfected with siRNA targeting *CCND1* and *CDK4*. Twenty-four hours after transfection, protein expression was analyzed (right) and cells were treated with 8 nM bortezomib. Cell death was assessed by AnnexinV-PI staining 24 h post-treatment (left). Data represent means ± S.D. from three independent experiments. (TIFF 833 kb)

Additional file 4: **Figure S3.** Double knockdown of cyclin D1 and CDK4 antagonizes bortezomib-induced cell death similar to single knockdown of CDK4. MCL cell line Mino was transfected with siRNA targeting CCND1, CDK4, or both. Twenty-four hours after transfection, protein expression was analyzed (right) and cells were treated with 8 nM bortezomib. Cell death was assessed by AnnexinV-PI staining 24 h post-treatment (left). Data represent means ± S.D. from three independent experiments. (TIFF 903 kb)

Additional file 5: **Figure S4.** CDK4 inhibition antagonizes cell death of NOXA inducing substances. MCL cell line Jeko-1 was pretreated with 300 nM palbociclib for 16 h and subsequently co-treated with either 8 nM carfilzomib, 40 μM orlistat, or 500 μM hydrogen peroxide. After 24 h treatment, cell death was assessed by AnnexinV-PI staining. Data represent means ± S.D. from three independent experiments. (TIFF 950 kb)

Additional file 6: **Figure S5.** Inhibition CDK4 activity hardly alters *NOXA* mRNA levels after proteasome inhibition. MCL cell line Mino was treated with 100 nM and Granta-519 with 300 nM Palbociclib for 16 h and subsequently co-treated with 8 nM bortezomib. After 8 h co-treatment samples were taken and analyzed by real-time PCR. *NOXA* mRNA expression was normalized to TBP. Data represent means ± SD from three experiments. (TIFF 569 kb)

Additional file 7: **Figure S6**. Palbociclib-mediated antagonism on bortezomib-induced cell death is not caused by alterations in cell cycle distribution. MCL cell line Mino was transfected with siRNA targeting RB1 and treated with 100 nM palbociclib 24 h post-transfection. After 16 h, cells were treated with 8 nM bortezomib. Twenty-four hours after treatment, cell cycle distribution was measured by BrdU staining (left), cell death was assessed by AnnexinV-PI staining (middle panel), and proteins were analyzed by Western blot (right). Data represent means ± S.D. from three independent experiments. (TIFF 802 kb)

Additional file 8: **Figure S7.** Palbociclib treatment induces autophagy but not after a short treatment period. (A) MCL cell line Jeko-1 was treated with 300 nM palbociclib for 24 h with or without 40 μM hydroxychloroquine. After treatment, autophagic vesicles were measured with Cyto-ID staining. (B) MCL cell line Mino was treated with 100 nM palbociclib for 6 h. After treatment autophagic vesicles were measured with Cyto-ID staining. (TIFF 1187 kb)

Additional file 9: **Figure S8.** Autophagy inhibitors counteract palbociclib-mediated antagonism on bortezomib-induced cell death. MCL cell line Jeko-1 was treated with 20 μM liensinine (left), 2 mM 3-MA (left), or 10 μM Spautin-1 (right) with or without 300 nM palbociclib. After 16 h, cells were treated with 8 nM bortezomib for 24 h and analyzed by AnnexinV-PI staining to assess cell death. Data represent means ± S.D. from three independent experiments. (TIFF 690 kb)

Additional file 10: **Figure S9.** Co-treatment of bortezomib with autophagy inhibitors potentiates cell death induction. MCL cell line Rec-1 was pretreated with 20 μM liensinine, 120 μM hydroxychloroquine, or 5 mM 3-MA for 16 h and subsequently co-treated with 8 nM bortezomib. After 24 h treatment, cell death was assessed by AnnexinV-PI staining. Data represent means ± S.D. from three independent experiments. (TIFF 725 kb)

Additional file 11: **Figure S10.** Synergistic cell death after proteasome inhibition and simultaneous fatty acid inhibition is caspase dependent. MCL cell line Jeko-1 was treated with 50 μM of the pan-caspase inhibitor Z-VAD-FMK for 2 h subsequently treated with 7 nM bortezomib or carfilzomib and co-treated with 15 μM orlistat. After 24 h, cell death was assessed by AnnexinV-PI staining. Data represent means ± S.D. from three experiments. (TIFF 774 kb)

Additional file 12: **Figure S11.** Combination of proteasome inhibition and simultaneous fatty acid inhibition regulates mainly NOXA protein levels and not PUMA, BAX, BAK, or MCL1. MCL cell line Jeko-1 was treated with 7 nM bortezomib or carfilzomib and co-treated with 15 μM orlistat. After 14 h, protein expression was analyzed by Western blot. (TIFF 1502 kb)

Additional file 13: **Figure S12.** Proteasome inhibitors combined with fatty acid inhibition induce synergistic cell death. MCL cell line Jeko-1 was treated with either five concentrations of carfilzomib or four concentrations of bortezomib and co-treated with four concentrations of orlistat (concentrations in the table). After 24 h, cell death was assessed by AnnexinV-PI staining. Induced cell death was used as fractional effect for determining the combination index (CI). (TIFF 1773 kb)

Additional file 14: **Figure S13.** NOXA protein contains a potential LIR motif. The amino acid sequence DGFRRL at the position 29-34 in the NOXA protein represents a potential LIR motif with the core consensus sequence ((W/F/Y) XX (L/I/V)). The acidic amino acid is highlighted in red. (TIFF 829 kb)

## Abbreviations

3-MA: 3-Methyladenine
ALP: Autophagy lysosome pathway
CDK4: Cyclin-dependent kinase 4
CI: Combination index
CMA: Chaperone-mediated autophagy
cosi: Control siRNA
FASNi: Fatty acid synthase inhibitor
MCL: Mantle cell lymphoma
PBMNC: Peripheral blood mononuclear cells
ROS: Reactive oxygen species
SD: Standard deviation
UPS: Ubiquitin proteasome
