# Supplementary figures and images for: Cyclin D1-CDK4 activity drives sensitivity to bortezomib in mantle cell lymphoma by blocking autophagy-mediated proteolysis of NOXA
Source: J Hematol Oncol. 2018 Sep 4;11:112. doi: 10.1186/s13045-018-0657-6 (PMC6123978; doi:10.1186/s13045-018-0657-6)

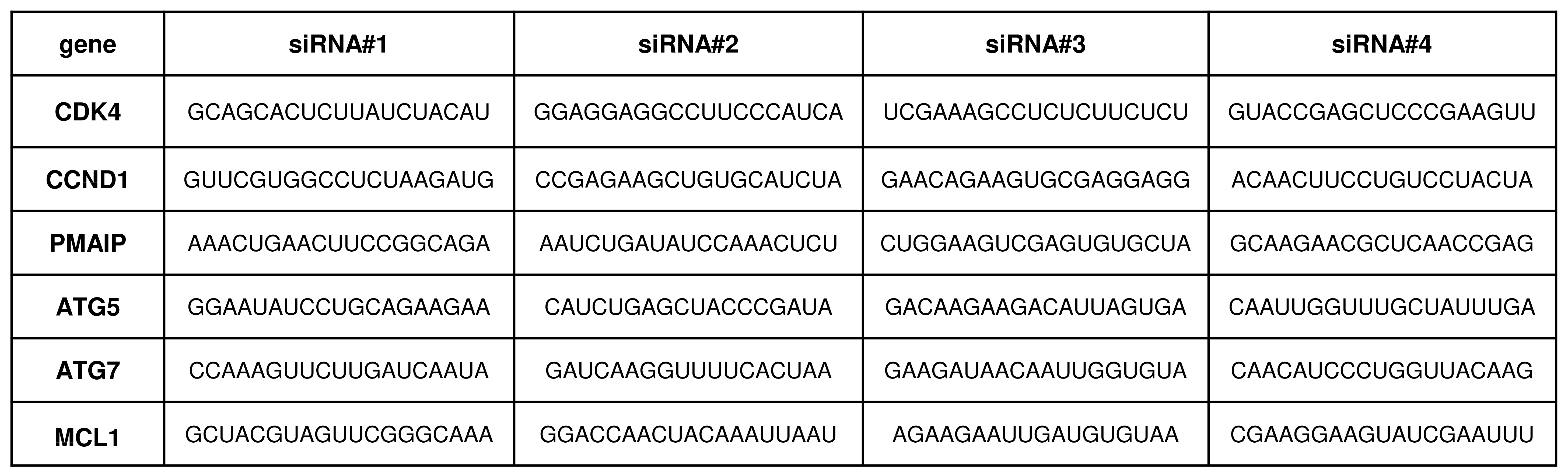

Supplement: Supplementary file 1 — Table S1. Sequences targeted by the siRNAs used for gene silencing. (TIFF 883 kb) [file 13045_2018_657_MOESM1_ESM.tiff]

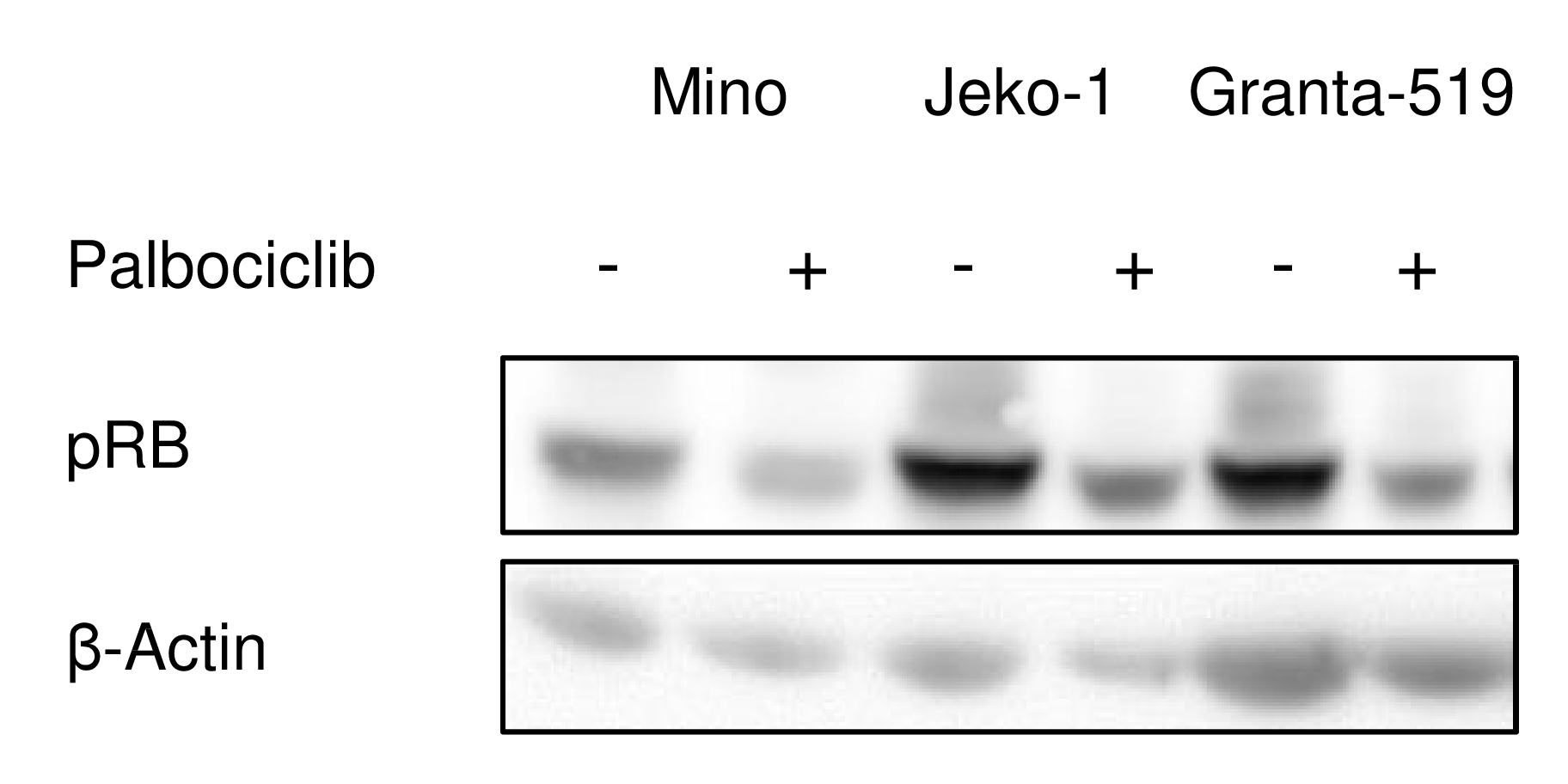

Supplement: Supplementary file 2 — Figure S1. CDK4 inhibition by palbociclib treatment inhibits RB1 phosphorylation. MCL cell lines Jeko-1 and Granta-519 were treated with 300 nM and MCL cell line Mino with 100 nM palbociclib. After 16 h, proteins were analyzed by Western blot. (TIFF 360 kb) [file 13045_2018_657_MOESM2_ESM.tiff]

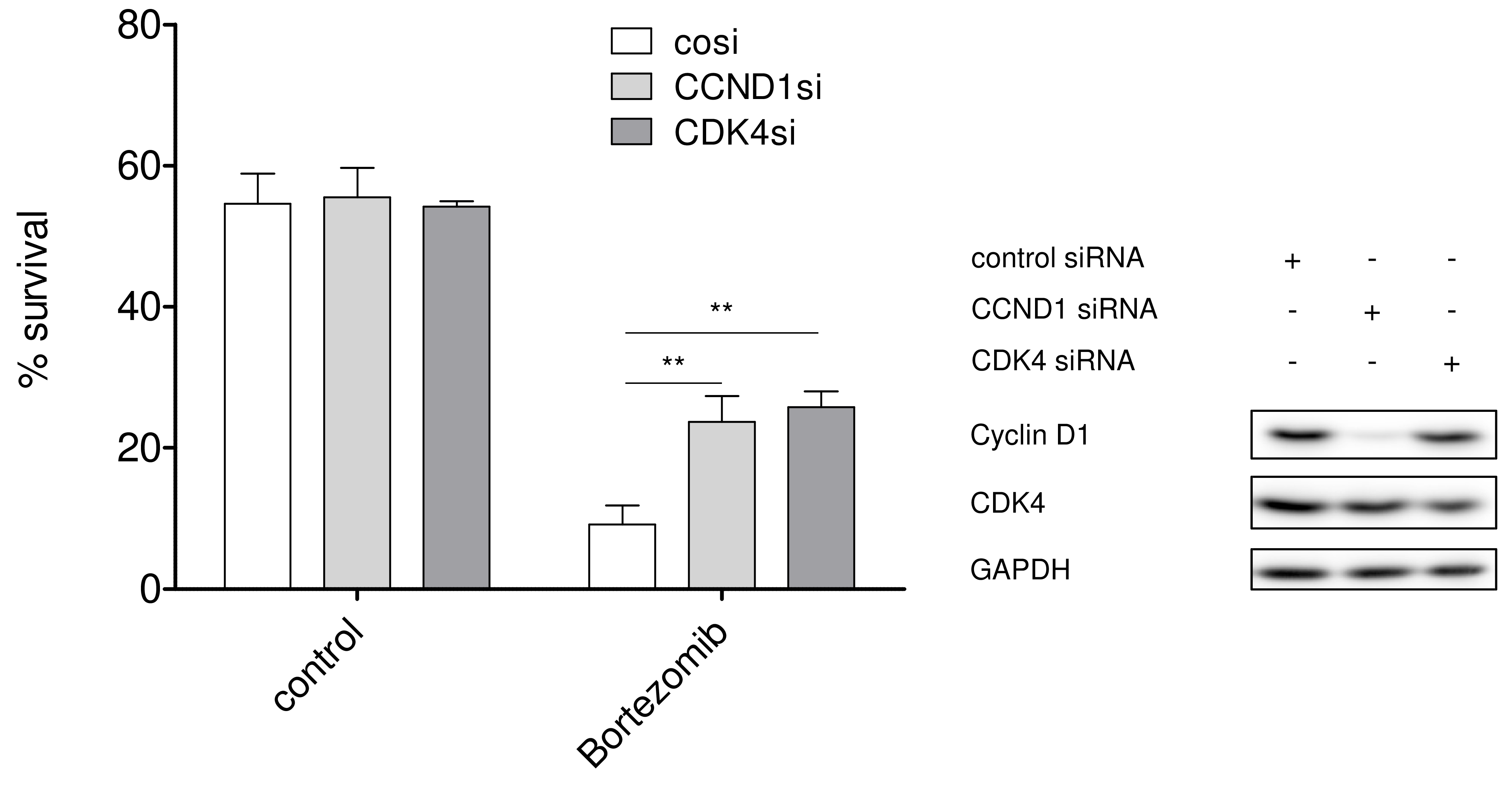

Supplement: Supplementary file 3 — Figure S2. Knockdown of cyclin D1 and CDK4 antagonizes bortezomib-induced cell death. MCL cell line Jeko-1 was transfected with siRNA targeting CCND1 and CDK4. Twenty-four hours after transfection, protein expression was analyzed (right) and cells were treated with 8 nM bortezomib. Cell death was assessed by AnnexinV-PI staining 24 h post-treatment (left). Data represent means ± S.D. from three independent experiments. (TIFF 833 kb) [file 13045_2018_657_MOESM3_ESM.tiff]

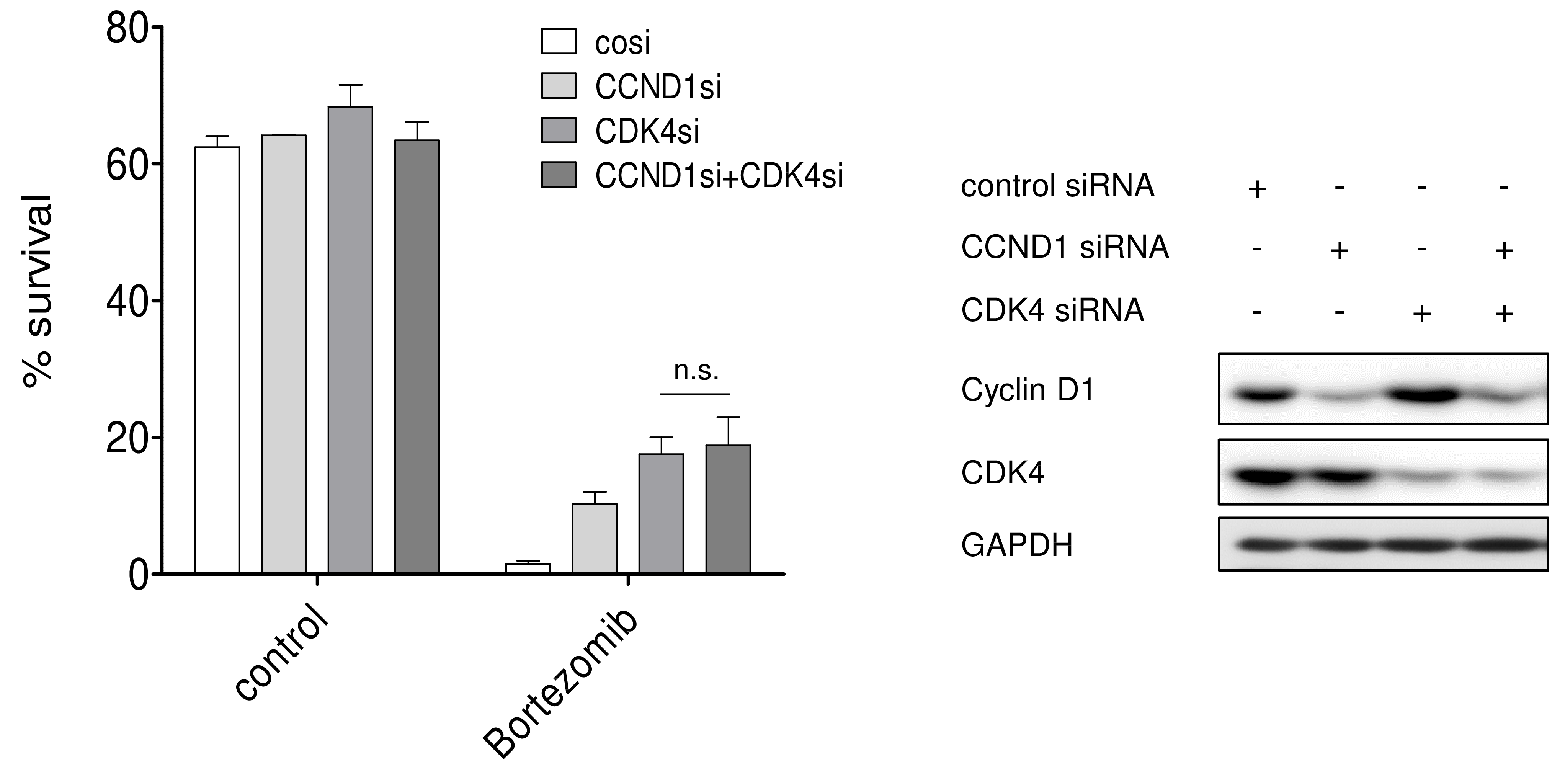

Supplement: Supplementary file 4 — Figure S3. Double knockdown of cyclin D1 and CDK4 antagonizes bortezomib-induced cell death similar to single knockdown of CDK4. MCL cell line Mino was transfected with siRNA targeting CCND1, CDK4, or both. Twenty-four hours after transfection, protein expression was analyzed (right) and cells were treated with 8 nM bortezomib. Cell death was assessed by AnnexinV-PI staining 24 h post-treatment (left). Data represent means ± S.D. from three independent experiments. (TIFF 903 kb) [file 13045_2018_657_MOESM4_ESM.tiff]

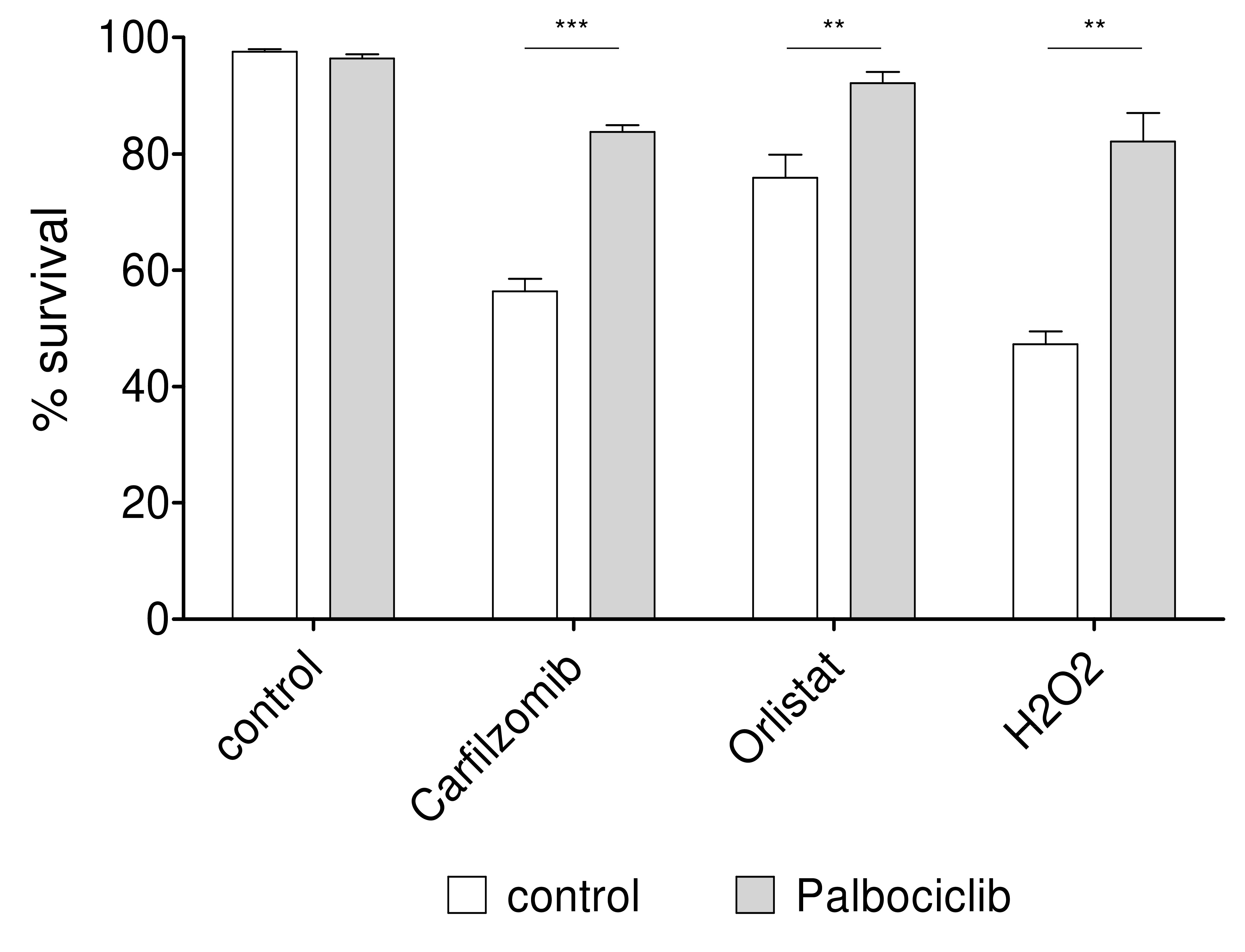

Supplement: Supplementary file 5 — Figure S4. CDK4 inhibition antagonizes cell death of NOXA inducing substances. MCL cell line Jeko-1 was pretreated with 300 nM palbociclib for 16 h and subsequently co-treated with either 8 nM carfilzomib, 40 μM orlistat, or 500 μM hydrogen peroxide. After 24 h treatment, cell death was assessed by AnnexinV-PI staining. Data represent means ± S.D. from three independent experiments. (TIFF 950 kb) [file 13045_2018_657_MOESM5_ESM.tiff]

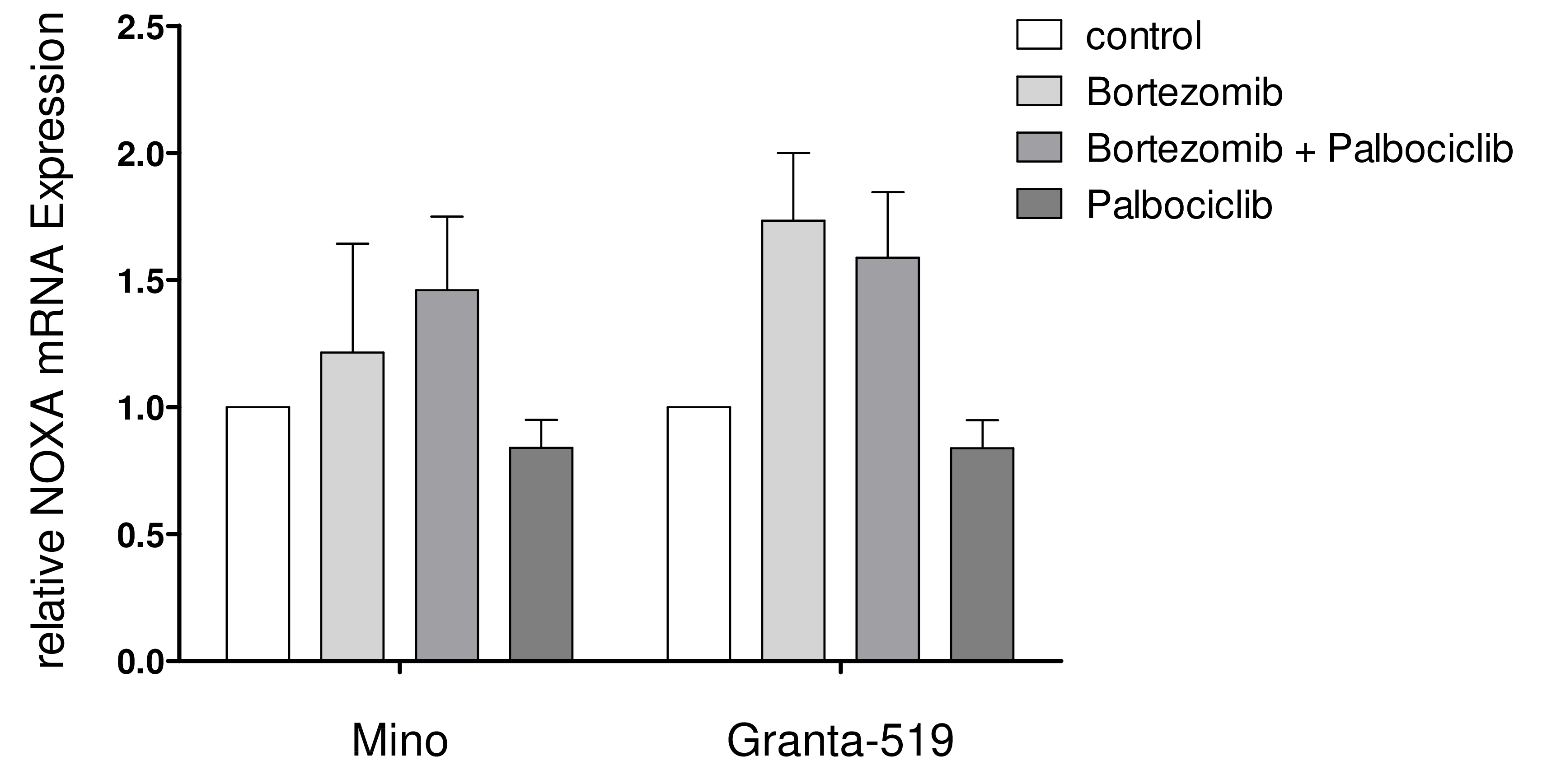

Supplement: Supplementary file 6 — Figure S5. Inhibition CDK4 activity hardly alters NOXA mRNA levels after proteasome inhibition. MCL cell line Mino was treated with 100 nM and Granta-519 with 300 nM Palbociclib for 16 h and subsequently co-treated with 8 nM bortezomib. After 8 h co-treatment samples were taken and analyzed by real-time PCR. NOXA mRNA expression was normalized to TBP. Data represent means ± SD from three experiments. (TIFF 569 kb) [file 13045_2018_657_MOESM6_ESM.tiff]

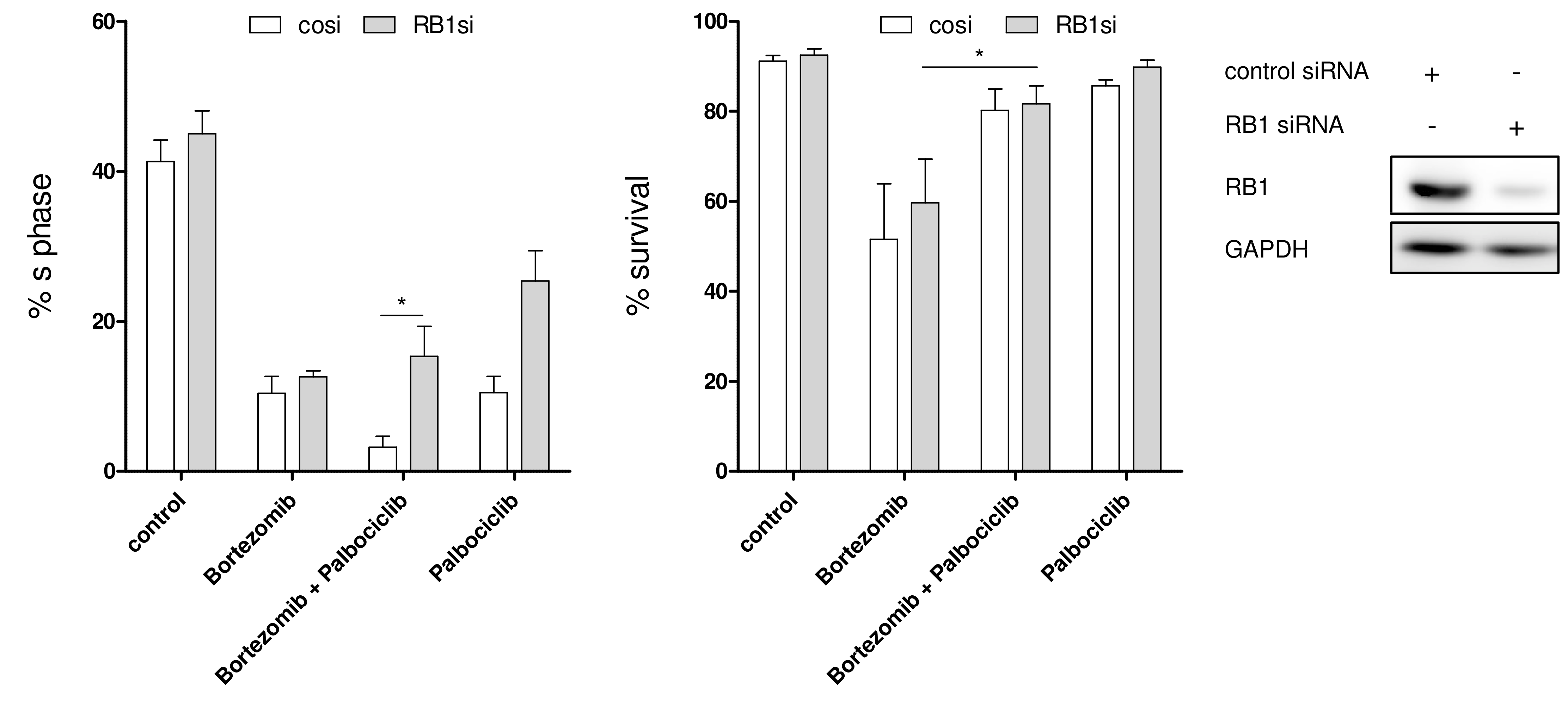

Supplement: Supplementary file 7 — Figure S6. Palbociclib-mediated antagonism on bortezomib-induced cell death is not caused by alterations in cell cycle distribution. MCL cell line Mino was transfected with siRNA targeting RB1 and treated with 100 nM palbociclib 24 h post-transfection. After 16 h, cells were treated with 8 nM bortezomib. Twenty-four hours after treatment, cell cycle distribution was measured by BrdU staining (left), cell death was assessed by AnnexinV-PI staining (middle panel), and proteins were analyzed by Western blot (right). Data represent means ± S.D. from three independent experiments. (TIFF 802 kb) [file 13045_2018_657_MOESM7_ESM.tiff]

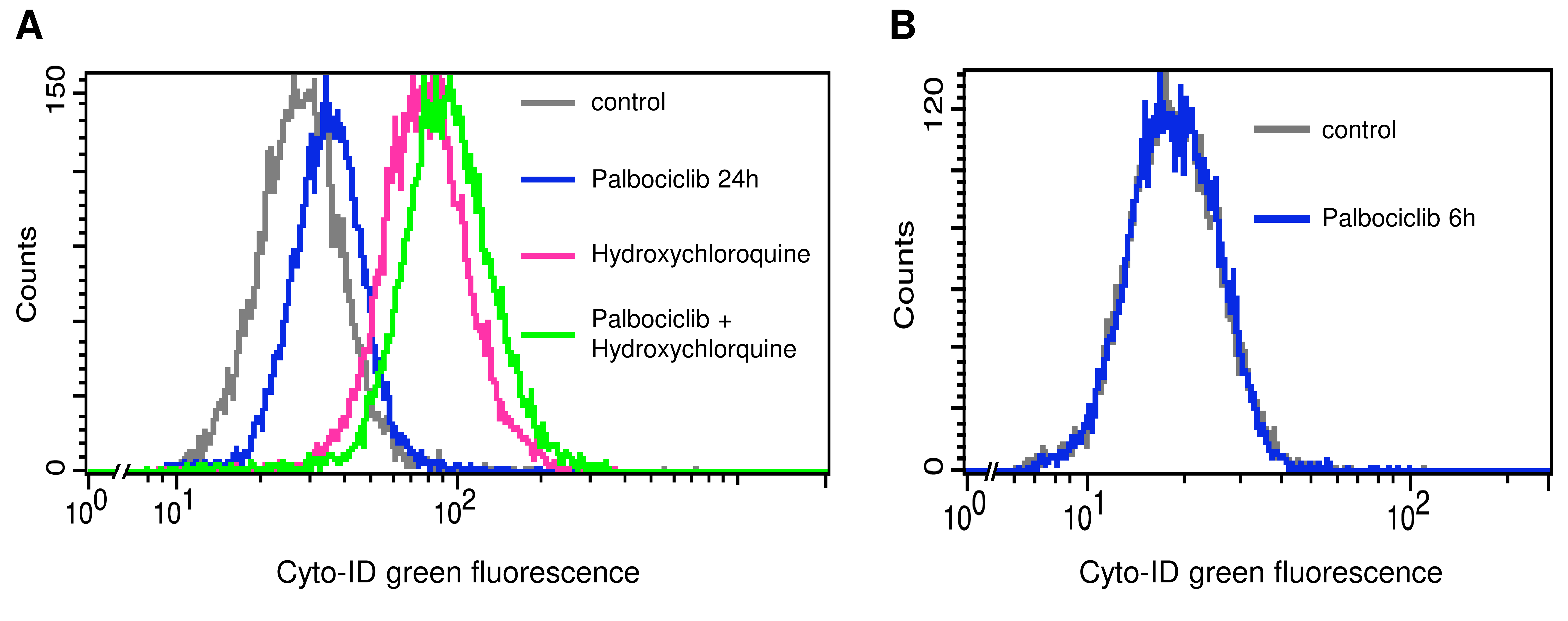

Supplement: Supplementary file 8 — Figure S7. Palbociclib treatment induces autophagy but not after a short treatment period. (A) MCL cell line Jeko-1 was treated with 300 nM palbociclib for 24 h with or without 40 μM hydroxychloroquine. After treatment, autophagic vesicles were measured with Cyto-ID staining. (B) MCL cell line Mino was treated with 100 nM palbociclib for 6 h. After treatment autophagic vesicles were measured with Cyto-ID staining. (TIFF 1187 kb) [file 13045_2018_657_MOESM8_ESM.tiff]

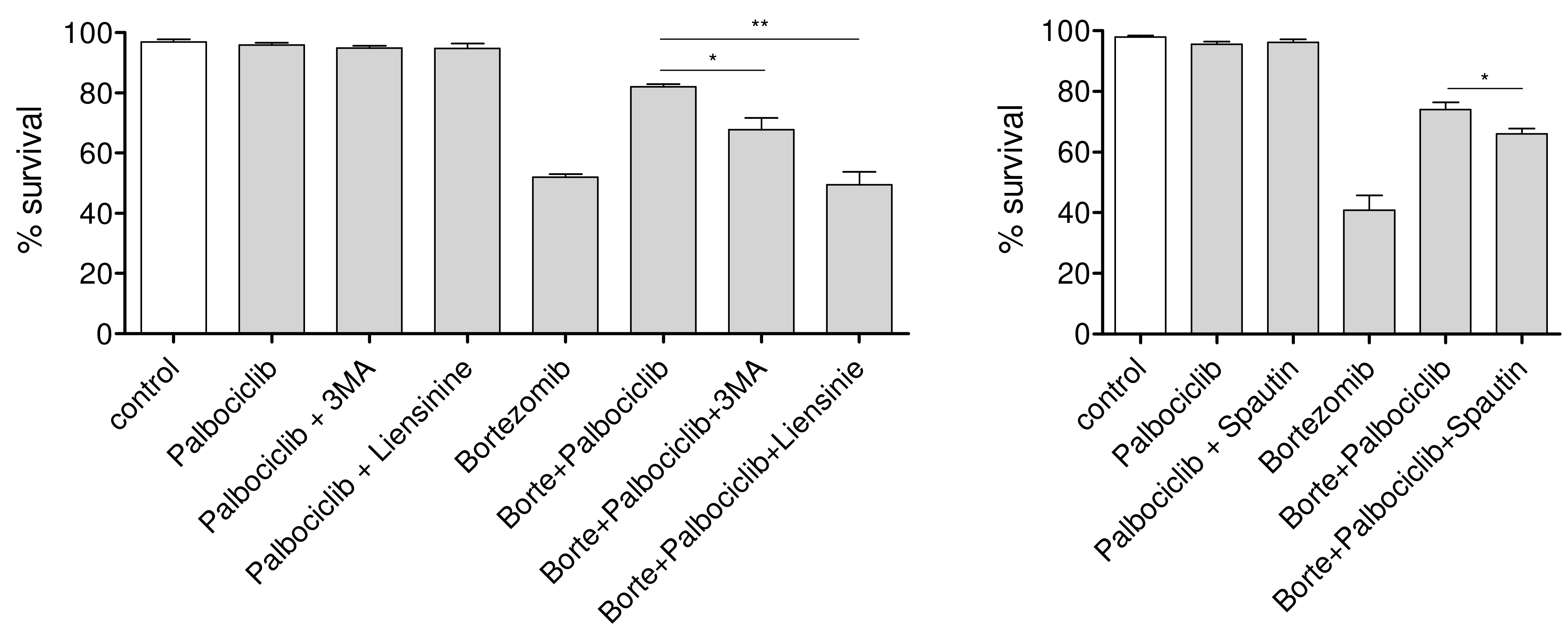

Supplement: Supplementary file 9 — Figure S8. Autophagy inhibitors counteract palbociclib-mediated antagonism on bortezomib-induced cell death. MCL cell line Jeko-1 was treated with 20 μM liensinine (left), 2 mM 3-MA (left), or 10 μM Spautin-1 (right) with or without 300 nM palbociclib. After 16 h, cells were treated with 8 nM bortezomib for 24 h and analyzed by AnnexinV-PI staining to assess cell death. Data represent means ± S.D. from three independent experiments. (TIFF 690 kb) [file 13045_2018_657_MOESM9_ESM.tiff]

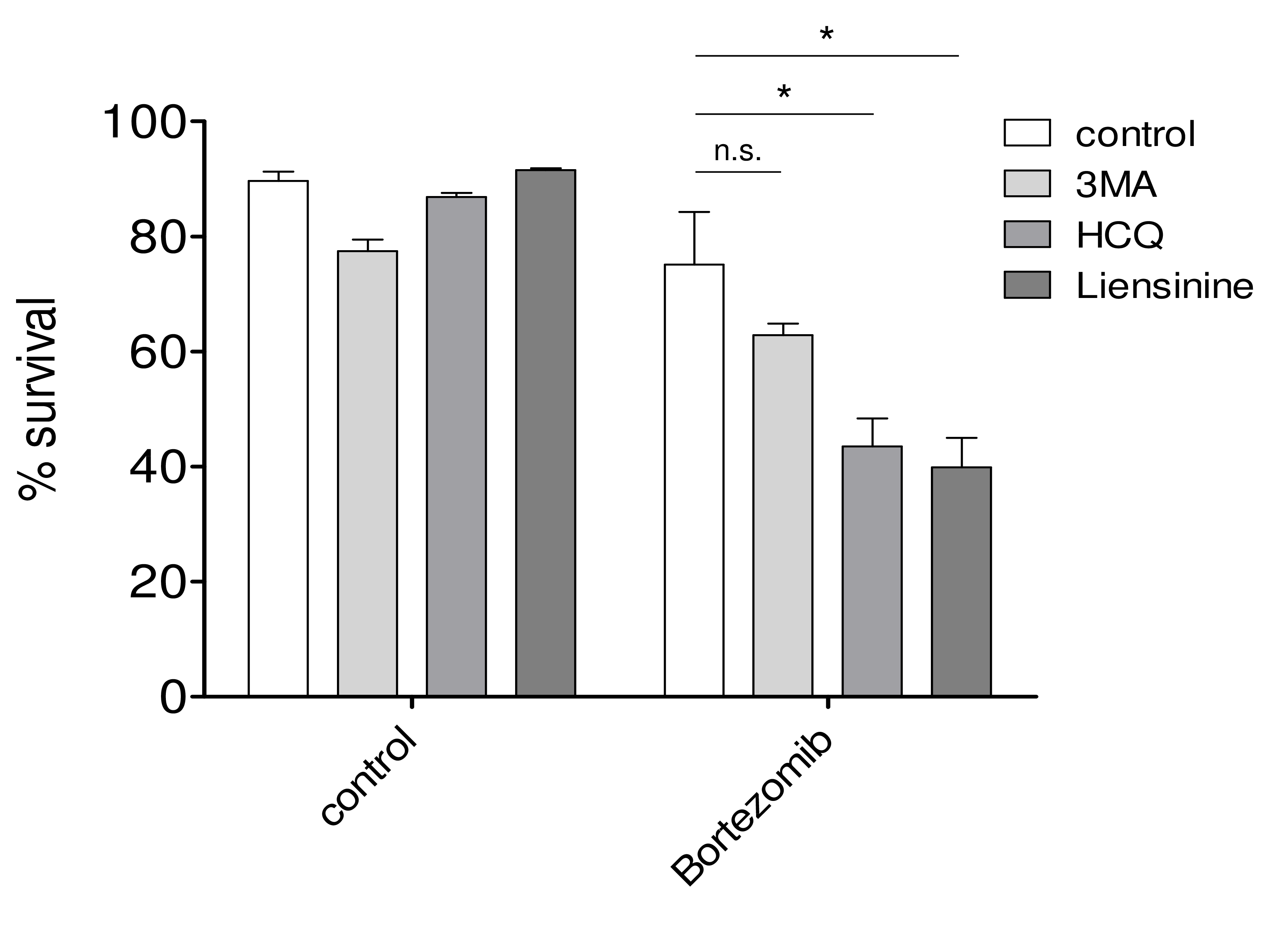

Supplement: Supplementary file 10 — Figure S9. Co-treatment of bortezomib with autophagy inhibitors potentiates cell death induction. MCL cell line Rec-1 was pretreated with 20 μM liensinine, 120 μM hydroxychloroquine, or 5 mM 3-MA for 16 h and subsequently co-treated with 8 nM bortezomib. After 24 h treatment, cell death was assessed by AnnexinV-PI staining. Data represent means ± S.D. from three independent experiments. (TIFF 725 kb) [file 13045_2018_657_MOESM10_ESM.tiff]

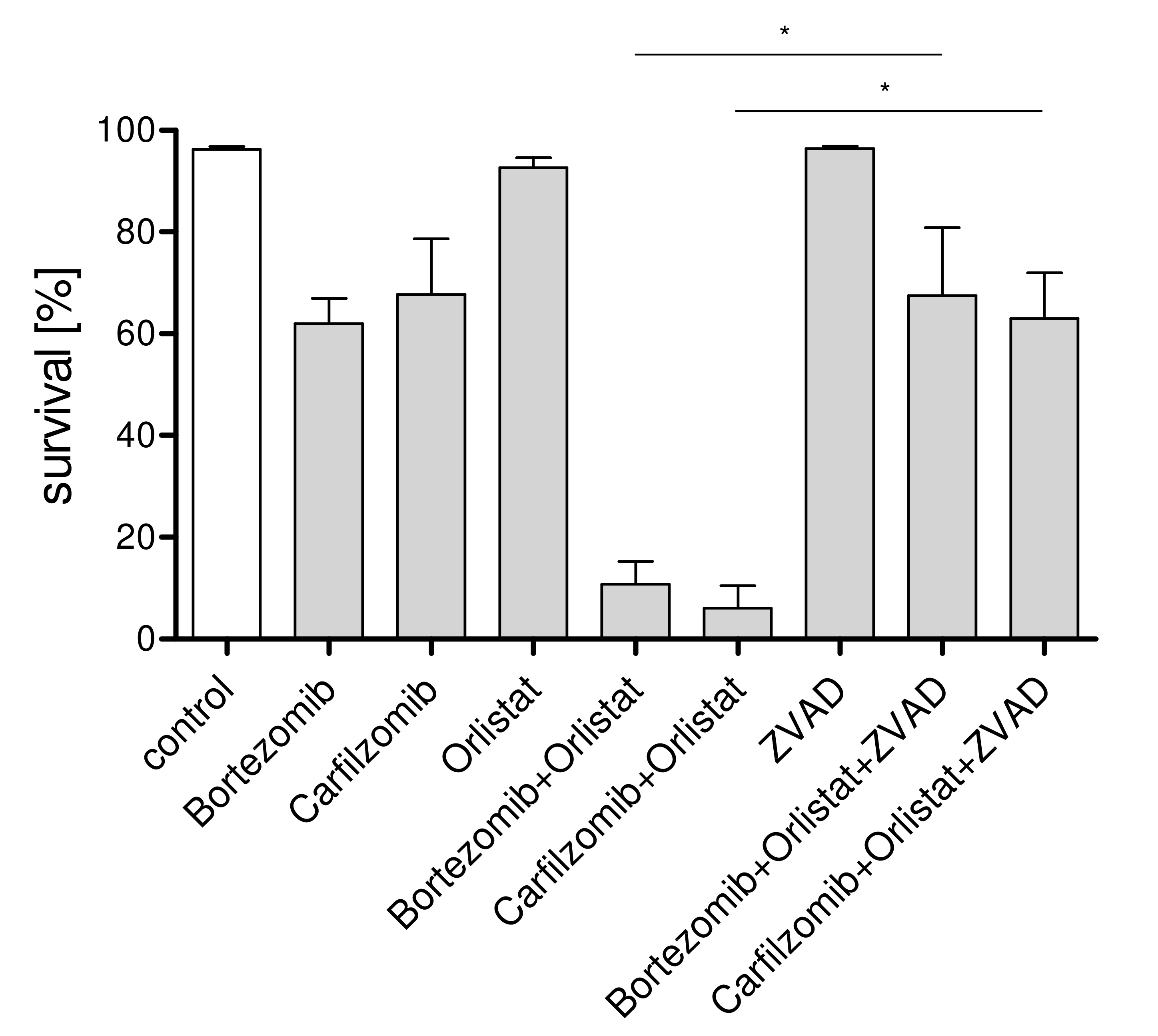

Supplement: Supplementary file 11 — Figure S10. Synergistic cell death after proteasome inhibition and simultaneous fatty acid inhibition is caspase dependent. MCL cell line Jeko-1 was treated with 50 μM of the pan-caspase inhibitor Z-VAD-FMK for 2 h subsequently treated with 7 nM bortezomib or carfilzomib and co-treated with 15 μM orlistat. After 24 h, cell death was assessed by AnnexinV-PI staining. Data represent means ± S.D. from three experiments. (TIFF 774 kb) [file 13045_2018_657_MOESM11_ESM.tiff]

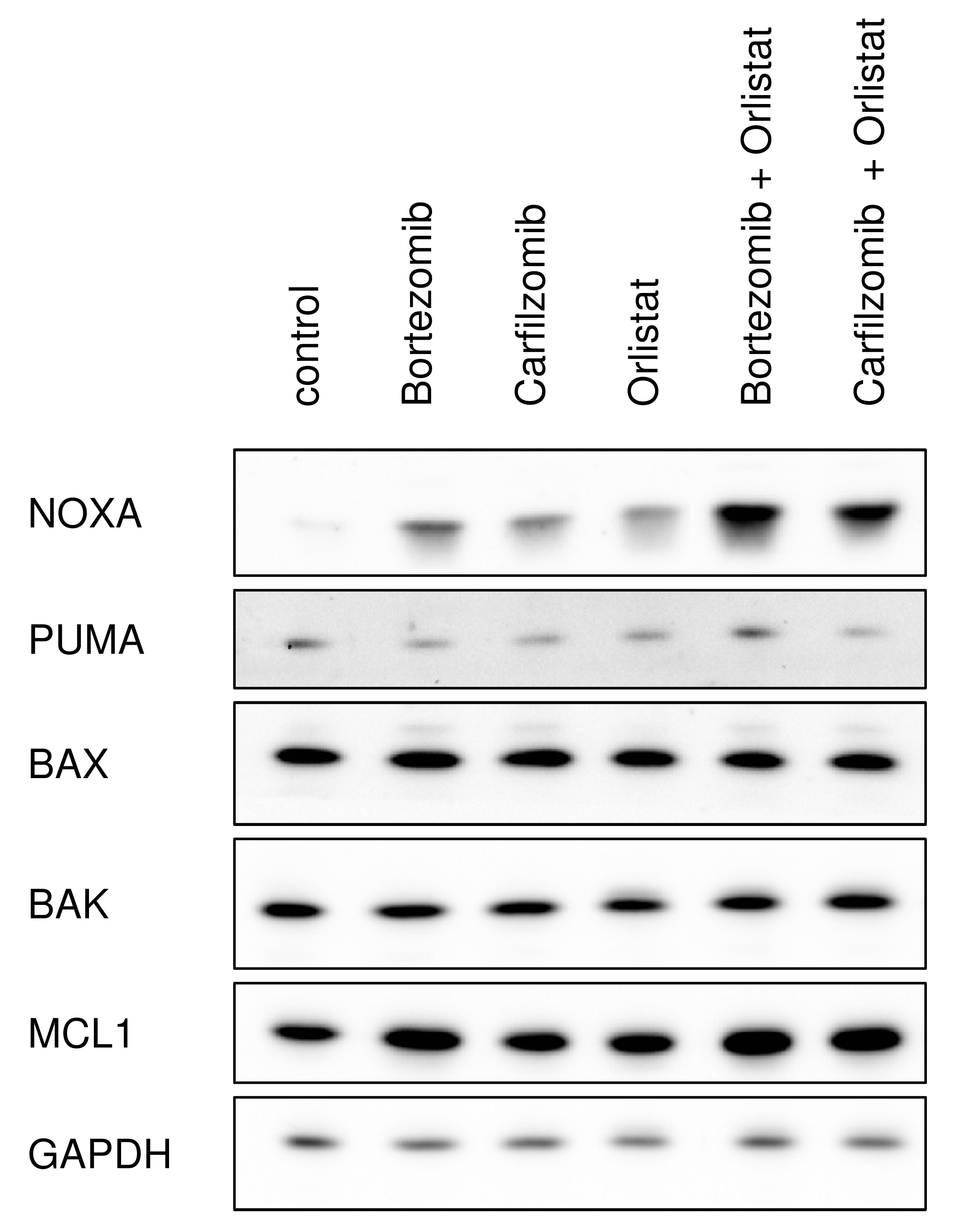

Supplement: Supplementary file 12 — Figure S11. Combination of proteasome inhibition and simultaneous fatty acid inhibition regulates mainly NOXA protein levels and not PUMA, BAX, BAK, or MCL1. MCL cell line Jeko-1 was treated with 7 nM bortezomib or carfilzomib and co-treated with 15 μM orlistat. After 14 h, protein expression was analyzed by Western blot. (TIFF 1502 kb) [file 13045_2018_657_MOESM12_ESM.tiff]

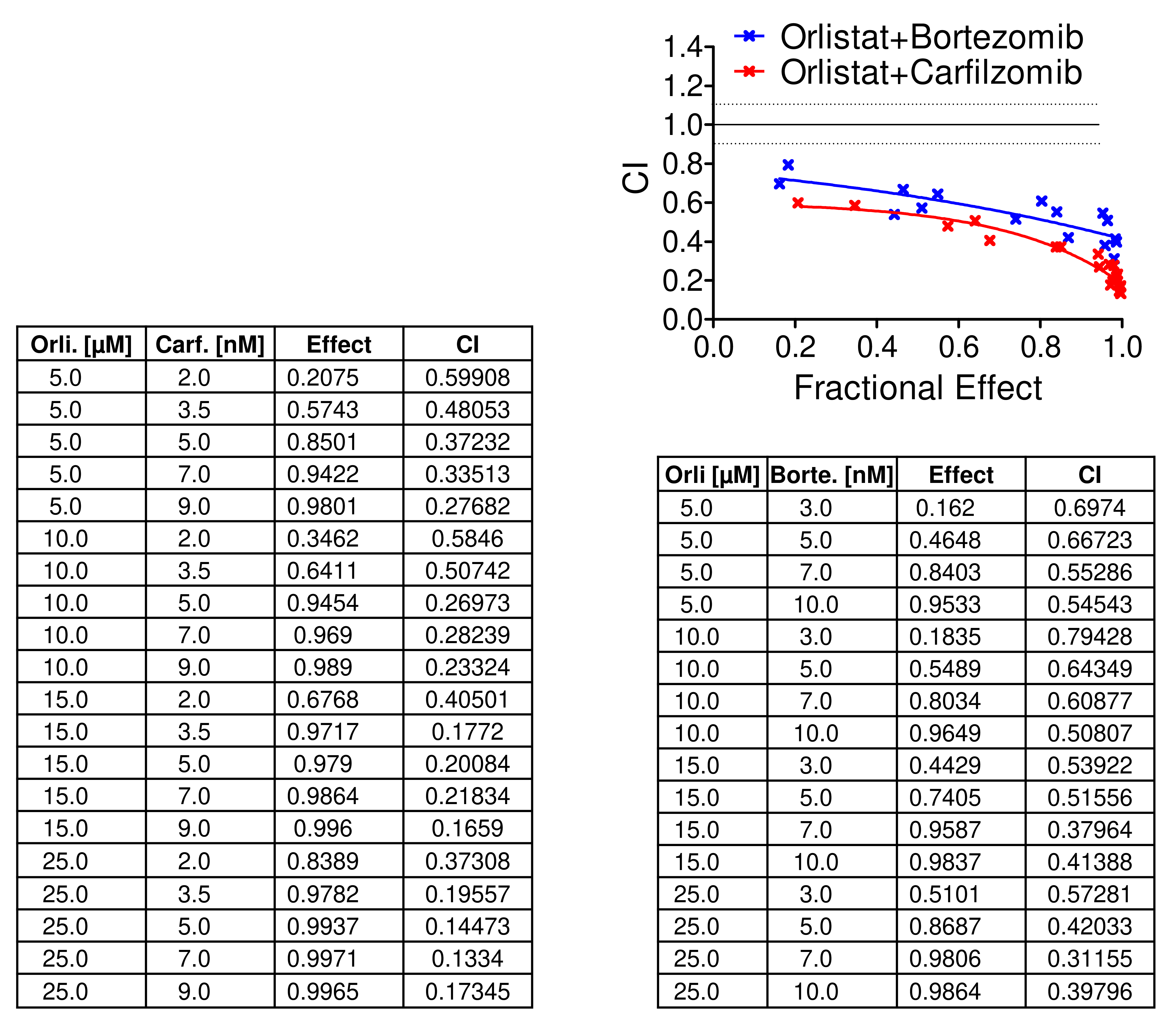

Supplement: Supplementary file 13 — Figure S12. Proteasome inhibitors combined with fatty acid inhibition induce synergistic cell death. MCL cell line Jeko-1 was treated with either five concentrations of carfilzomib or four concentrations of bortezomib and co-treated with four concentrations of orlistat (concentrations in the table). After 24 h, cell death was assessed by AnnexinV-PI staining. Induced cell death was used as fractional effect for determining the combination index (CI). (TIFF 1773 kb) [file 13045_2018_657_MOESM13_ESM.tiff]

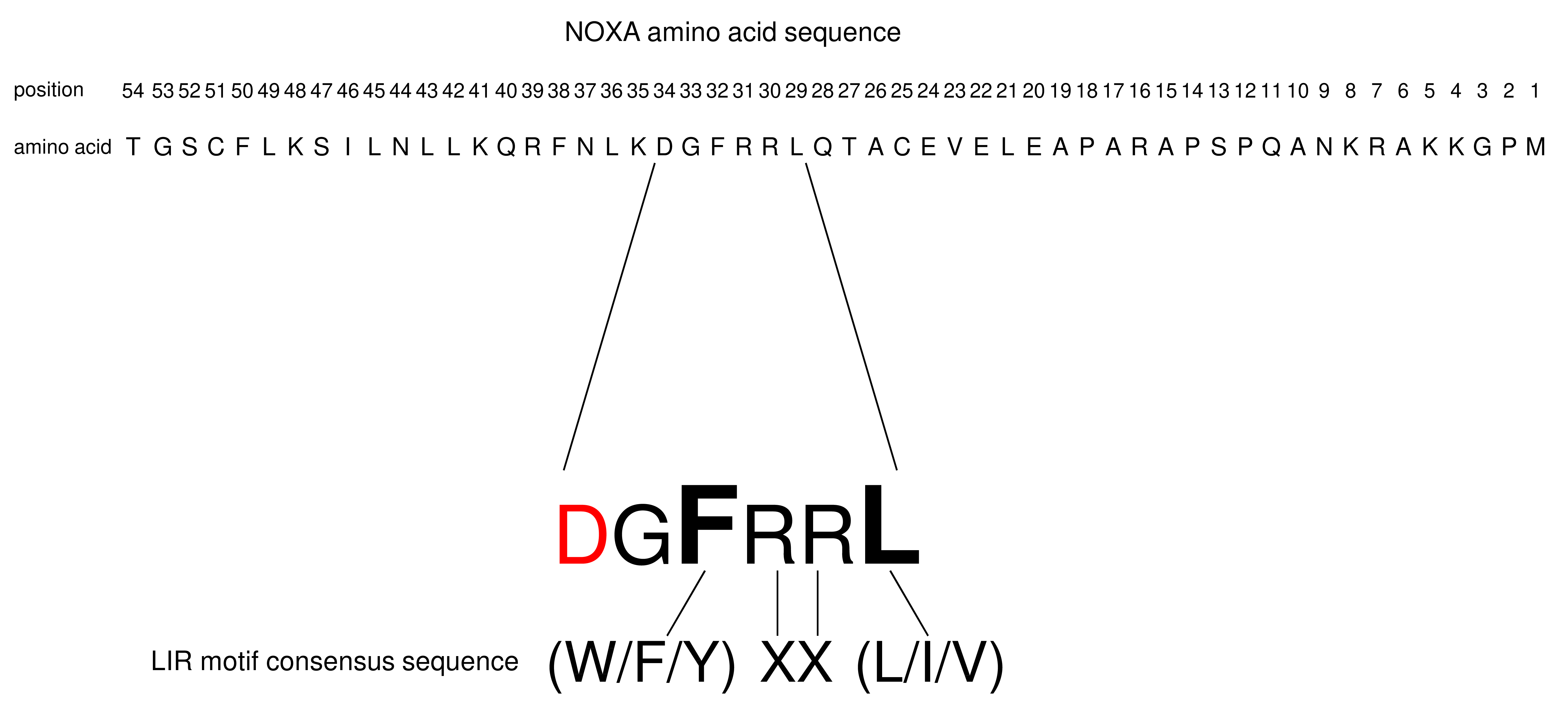

Supplement: Supplementary file 14 — Figure S13. NOXA protein contains a potential LIR motif. The amino acid sequence DGFRRL at the position 29-34 in the NOXA protein represents a potential LIR motif with the core consensus sequence ((W/F/Y) XX (L/I/V)). The acidic amino acid is highlighted in red. (TIFF 829 kb) [file 13045_2018_657_MOESM14_ESM.tiff]
